# MFCIS: an automatic leaf-based identification pipeline for plant cultivars using deep learning and persistent homology

**DOI:** 10.1038/s41438-021-00608-w

**Published:** 2021-08-01

**Authors:** Yanping Zhang, Jing Peng, Xiaohui Yuan, Lisi Zhang, Dongzi Zhu, Po Hong, Jiawei Wang, Qingzhong Liu, Weizhen Liu

**Affiliations:** 1 School of Computer Science and Technology, Wuhan University of Technology, Wuhan, Hubei China; 2 Chongqing Research Institute, Wuhan University of Technology, Chongqing, China; 3 Shandong Key Laboratory of Fruit Biotechnology Breeding, Shandong Institute of Pomology, Taian, Shandong China

**Keywords:** Plant breeding, Field trials, Biodiversity, Bioinformatics

## Abstract

Recognizing plant cultivars reliably and efficiently can benefit plant breeders in terms of property rights protection and innovation of germplasm resources. Although leaf image-based methods have been widely adopted in plant species identification, they seldom have been applied in cultivar identification due to the high similarity of leaves among cultivars. Here, we propose an automatic leaf image-based cultivar identification pipeline called MFCIS (Multi-feature Combined Cultivar Identification System), which combines multiple leaf morphological features collected by persistent homology and a convolutional neural network (CNN). Persistent homology, a multiscale and robust method, was employed to extract the topological signatures of leaf shape, texture, and venation details. A CNN-based algorithm, the Xception network, was fine-tuned for extracting high-level leaf image features. For fruit species, we benchmarked the MFCIS pipeline on a sweet cherry (*Prunus avium* L.) leaf dataset with >5000 leaf images from 88 varieties or unreleased selections and achieved a mean accuracy of 83.52%. For annual crop species, we applied the MFCIS pipeline to a soybean (Glycine max L. Merr.) leaf dataset with 5000 leaf images of 100 cultivars or elite breeding lines collected at five growth periods. The identification models for each growth period were trained independently, and their results were combined using a score-level fusion strategy. The classification accuracy after score-level fusion was 91.4%, which is much higher than the accuracy when utilizing each growth period independently or mixing all growth periods. To facilitate the adoption of the proposed pipelines, we constructed a user-friendly web service, which is freely available at http://www.mfcis.online.

## Introduction

Plant cultivar identification is a fundamental field of interest for plant breeding in terms of cultivar rights protection and the continuous breeding of new cultivars. The conventional method for cultivar identification usually relies on the expertize and empirical knowledge of crop breeders and has unpredictable accuracy and low efficiency. In recent decades, diverse molecular markers have been employed for plant variety identification^1–4^. Nevertheless, the accuracies of these methods mainly depend on the amount of genetic differentiation across the cultivars evaluated and the number and type of molecular markers selected in the analysis system^2^. These methods involve complicated operating procedures in a laboratory that are time-consuming and are not well suited for use by ordinary farmers. Therefore, rapid, stable, and accurate methods for plant cultivar identification are required.

A computer vision pipeline based on morphological features extracted from leaf images is an ideal option. Compared with the other organs, plant leaves have more general and lasting characteristics in terms of shape, texture, and venation and therefore have been successfully used for plant species identification/plant taxonomy^5,6^. To date, many image processing-based algorithms and software, such as Leaf GUI^7^, Rosette Tracker^8^, Leaf-GP^9^, MorphoLeaf^10^, and Phenotiki^11^, that measure leaf morphological phenotypes have been reported. However, a majority of the phenotypes extracted using these algorithms are not descriptive enough to discriminate leaves from different cultivars. Moreover, there are some leaf image-based plant species identification pipelines that use features of leaf shape, texture, and venation independently^12–15^ or combine them^16–19^. Unfortunately, when transferring these methods from species identification to cultivar identification, most of them failed due to the high similarity of leaves among different cultivars. Recently, a deep convolutional neural network (DCNN)-based identification method for apple cultivars was reported and tested on an apple leaf image dataset consisting of more than 10,000 images from 14 cultivars^20^. The unprecedented accuracy demonstrated the fantastic effectiveness of CNNs in cultivar recognition. However, the DCNN-based method required a large number of leaf images for each cultivar to train the model. A backpropagation neural network-based cultivar identification method was proposed for oleander identification. Using 18 global leaf characters (morphology and color) extracted from 22 cultivars, it only achieved an average accuracy of approximately 54.55%^21^. Its accuracy might be improved by adding more local leaf features into the model, but the computation speed would be sacrificed. The multi-orientation region transform method was developed to characterize leaf contour and vein structure features simultaneously for soybean cultivar identification^22^. They achieved an accuracy of ~54.2% in the recognition of 100 soybean cultivars. This study was the first to investigate large-scale cultivar classification with leaf images and proved the effectiveness of leaf venation features in cultivar classification. However, the drawback is that it requires vein images with primary and secondary vein annotations, which is quite labor intensive.

These challenges of leaf image-based cultivar identification models motivated us to develop a cultivar identification pipeline with the following characteristics: (1) Automation: the leaf images can be directly fitted as the input into the pipeline, and the cultivar identification results are the output. (2) Multi-feature combination: the leaf morphological features are comprehensively measured both locally and globally to improve the identification accuracy. (3) High efficiency: the method does not require a large sample size for model training and has an acceptable computation time. (4) Generalization capacity: the model can be easily adapted to other plants. Therefore, we incorporated persistent homology (PH), a topological method, to capture the morphological signatures of leaf shape, texture, and venation both locally and globally. Topology is a branch of mathematics that concerns spatial properties under deformation while disallows tearing apart or reattaching^23,24^. The core idea of PH is to track the appearance and disappearance of holes in topological space at multiple scales. The obtained features are a set of points in the plane, called the persistence diagram^25^ (PD), which can characterize structural properties and reflect spatial information in an invariant way^26^. Compared to the traditional feature extractors and CNN-based methods, PH is more resilient to local perturbations and scale variance^25,27^. This is because although the shapes of connected components and holes may change under geometric transformations, the number of components and holes remains the same^28^. Therefore, it is recognized that the incorporation of morphological features extracted by PH into CNN-based models can provide a significant performance improvement. These ideas have been validated in a wide range of computer vision and biomedical image processing studies, such as automated tumor segmentation^29,30^, 3D surface texture analysis^31^, and image classification^27^.

In this study, we propose a leaf image-based and automatic plant cultivar classification pipeline, called MFCIS (Multi-feature Combined Cultivar Identification System), that combines morphological features of the leaf shape, texture, and venation extracted by PH and the high-level image features extracted by the fine-tuned Xception^32^ model. We primarily tested the proposed pipeline on a sweet cherry leaf cultivar dataset with >5000 leaf images at the same growth stage derived from 88 commercial cultivars or unreleased selections. To assess the generalization ability of the proposed pipeline, we also applied the MFCIS to a soybean leaf cultivar dataset consisting of 5000 leaf images of 100 cultivars. Unlike the sweet cherry dataset, leaves in the soybean dataset were collected from five different growth periods. Therefore, we attempted cultivar identification using leaves from each period independently, mixed leaves from all growth periods without taking the soybean growth periods into account, and leaves from each period independently with a fusion of the prediction results.

## Materials and methods

### Plant materials

#### Sweet cherry cultivar dataset

**Fig. 1:**
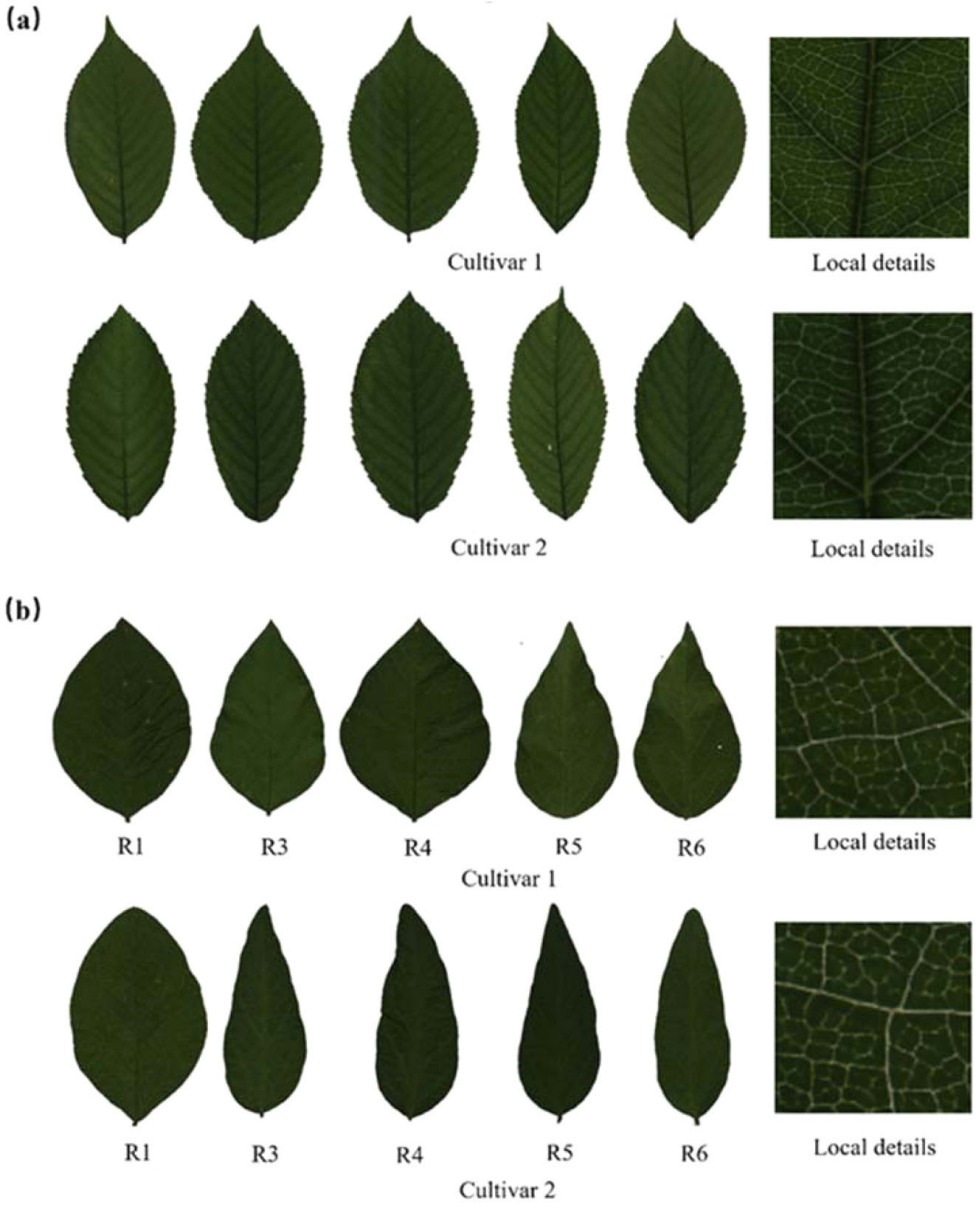
Examples of transmitted light leaf images. **a** Sweet cherry leaves of two cultivars. **b** Soybean leaves of two cultivars at five growth stages. The first and second rows contain two different cultivars. Columns one to five represent the growth periods of R1, R3, R4, R5, and R6, respectively. Images in the last column show the local details of the augmented leaf regions

Sweet cherry leaves were collected from 88 sweet cherry cultivars (64 commercial cultivars and 24 unreleased selections) at the research station of the Shandong Institute of Pomology in Tai’an, Shandong Province, China in May 2020. For each cultivar, we randomly sampled 50 to 90 mature leaves (Fig. S1) in the middle parts of the branches from 3 to 5 trees to ensure that these leaves were at the same growth stage. These leaves were imaged by transmission scanning (Fig. 1a) with a resolution of 600 DPI and color depth of 48 bits with an EPSON Perfection V850 Pro scanner (Epson Co., Ltd, Shanghai, China).

#### Soybean cultivar dataset

Leaves of 100 soybean cultivars were collected at the Soybean Experimental Station of Heilongjiang Academy of Agricultural Sciences in Mudanjiang, Heilongjiang Province, China, in 2017. For each cultivar, a sample of 50 fully expanded leaves was randomly picked from several plants at five reproductive stages: the beginning flowering, beginning pod, full pod, beginning seed, and full seed stages (denoted as R1, R3, R4, R5, and R6, respectively). These leaves were immediately scanned after they were detached from the plants using a backside transmission-type scanner (EPSON Perfection V850 Pro) under the same configuration as the sweet cherry leaf image dataset. Therefore, 5,000 leaf images were collected for our soybean cultivar dataset (Fig. 1b).

### Image analysis pipeline for cultivar identification

**Fig. 2:**
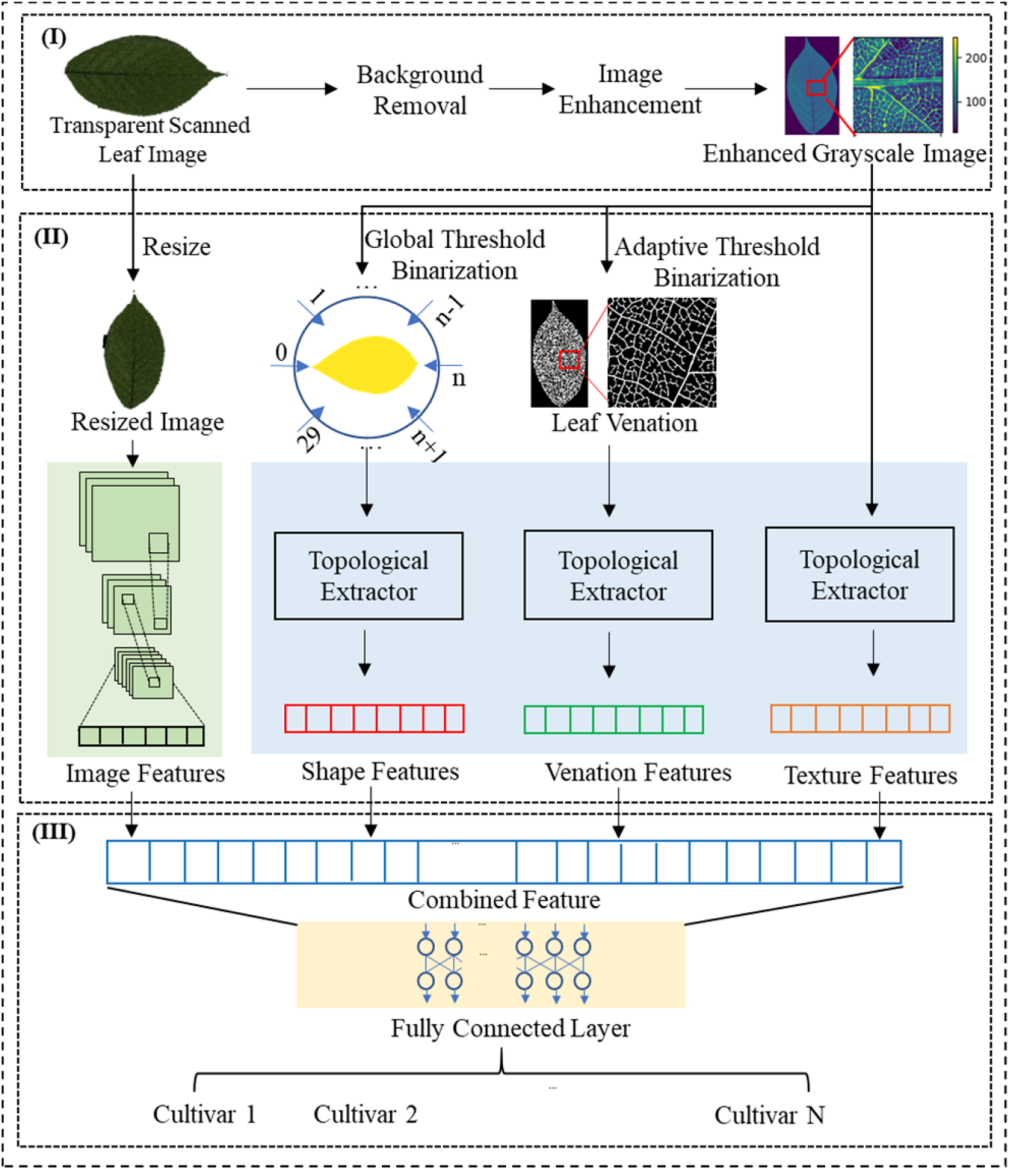
Flowchart for the proposed MFCIS method. **I** Data preprocessing. **II** Feature extraction using the persistent homology and CNN. **III** Multi-feature combination and cultivar identification

We developed a robust pipeline for automatically identifying cultivars using leaf images as input. A flowchart of the main algorithm is presented in Fig. 2 and is described in detail as follows: (I) Data preprocessing. A leaf RGB image was converted to a grayscale image, and the image was enhanced by extending the dynamic range of the grayscale value. (II) Feature extraction. Shape features were extracted by filtrating the leaf from different directions. Global leaf texture and venation features were captured by computing the PDs of the enhanced grayscale images and leaf venation images. PDs of the leaf shape, texture, and venation were further processed by a 1-D CNN model. Additional image features were extracted by a CNN model. (III) Feature combination and cultivar classification. The ‘early fusion’ technique^5^ was used to concatenate the leaf morphological features and the high-level CNN features. Crop cultivars were classified by a fully connected network using the combined features.

#### Image preprocessing

The image preprocessing procedure consists of background removal and contrast enhancement. Owing to the high quality and clean background of our leaf images, a typical global thresholding method was adopted to remove the image background, and linear stretching was applied to enlarge the dynamic grayscale value range.

#### Leaf morphological feature extraction using PH

As shown in Fig. 1, leaves of different cultivars exhibit distinct leaf morphological patterns in terms of their shapes, textures, and venations. Leaves of the same cultivar at different growth stages also display subtle differences. Hence, we employed PH to capture leaf shape, texture, and vein features from a topological perspective and described the extracted features with PDs. The leaf shape PDs were calculated with the Pershombox package^33^, and the leaf texture and venation PDs were both computed with the Python Homcloud package [Ippei Obayashi, Hiraoka lab (AIMR, Tohoku University)].

The detailed feature extraction procedure is described in supplementary materials section 1 and Fig. S2. Here, we briefly introduce the design philosophy. Inspired by Hofer et al.^33^ and Turner et al.^34^, the leaf shape features were analyzed by multi-directional height function filtration. We constructed the height functions for the leaf binary mask image from 30 directions, filtered these functions and stacked the PDs of the three consecutive directions to preserve the morphology context information. The light transmittance difference in leaf venation and mesophyll caused the unique textures in the transmission scanned images is indicated by the variety of grayscale values in the different leaf regions. Therefore, we formulated the leaf grayscale values as real value functions and filtered them by increasing the threshold from −∞ to +∞. In the filtration procedure, the changes in the components and holes were captured and recorded. For venation feature extraction, we applied the distance transform on binary leaf venation images and tracked the areola and venation structure changes under the different scales by filtrating the obtained distance map of the leaf venation network.

#### High-level image feature extraction by a CNN

The deep CNN model, Xception^32^, was implemented as the backbone network to extract high-level features from leaf images. It was difficult to train such a deep neural network from scratch on our relatively small dataset. Additionally, the knowledge learned from other image classification tasks may be helpful in our study. Therefore, we applied transfer learning to the cultivar classification task by adopting the weights of Xception on the ImageNet dataset^35^ as the initial settings. The output of the global average pooling layer is a 2048-dimensional vector and was used as the high-level image features for the following multifeature combination process. High-level image features are more abstract and discriminative than traditional hand-crafted morphological features (the leaf area, perimeter, etc.) and the image processing-based descriptors.

#### PD feature vectorization and multifeature combination

Since a PD is a set of points in a plane with unusual multiset structures, it is a considerable challenge to use it for machine learning purposes^33^. Here, our innovation is to vectorize the PDs of the leaf shape, texture, and venation by inputting them into a CNN. Compared to the other vectorization methods (such as persistence image^36^ and persistence landscape^37^), the input layer maps the PD features to a task-optimal representation with trainable weights^33^. Owing to the high definition and resolution of the transmitted scanned leaf images, the texture and venation PDs contain thousands of points with different distances from the diagonal, representing their ‘lifetime’. Empirically, features with a longer lifetime are usually more essential^27^, and noise generates points close to the diagonal. To balance the model complexity and accuracy, we sorted these points by their lifetime and selected the first 700 points for shape features and 1000 points for texture and venation features as input.

The topological features were further processed by a CNN model based on the previously mentioned input layer. The model structure was similar to the network for 2D object shape classification^33^, except for the filters and dropout layers. The network structure details are given in Fig. S3. There were ten layers in the topological feature model. The first layer was the input layer. For shape features, the PDs from three consecutive filtration directions were stacked as a 3-channel input. The number of filtration directions determined the branch number. The texture and venation features occupied four input branches. Both the 0th and 1st PD were used as input. A total of 16 filters were used in the following convolutional layers, and each of them learned different feature types. Each filter was slid over the input, and convolution was applied. The max-pooling layer reduced the feature dimension. It should be noted that max-pooling operated along the filter dimension. Dropout layers were used to avoid overfitting. The batch normalization layer was used to normalize the output of the previous layers. The ReLU layer performed a nonlinear transformation on the output of the previous layers.

To enhance the accuracy, we combined the outputs of the topological feature processing model and the Xception model. The concatenated feature was a 10,752-dimensional vector. The output dimension of the Xception model was 2048, and the dimension of the processed topological features was 8704; these processed features consisted of 30 shape feature streams, two texture feature streams, and two venation feature streams. A dropout layer followed the concatenation layer and had a dropout rate of 0.5, a dense layer with 2048 neurons, a batch normalization layer, and an output layer whose neuron number was the same as that of the image class.

#### Score-level fusion of the results derived from different growth periods

Leaves from different growth periods of the same crop cultivar may have different shapes, textures, and venation patterns, as shown in Fig. 1a. Leaves from different growth periods of the same cultivar may provide different but complementary clues for cultivar classification^38^. Therefore, we adopted a score-level fusion method to combine the prediction results of each growth period. The results were fused with (1).

1 $$G(X) = arg \,max \frac{1}{T}\mathop {\sum}\limits_{t = 1}^T {g_t(x_t)} ,\,X = \left\{ {x_1,x_2,...,x_T} \right\}$$

where *G(X)* is the final prediction result of the sample set *X*, *T* is the total model number, and *g*_*t*_(*x*_*t*_) is the prediction result of sample *x*_*t*_ by model *t*.

### Model evaluation

We evaluated our model performance with ten different train-and-test splits to ensure that the proposed pipeline is reliable and stable. In each iteration, the dataset was randomly shuffled and divided into training and test sets. There were ten iterations for each model, and the mean result was used to evaluate the model performance.

### Statistical analysis

We employed the one-way statistical analysis of variance (ANOVA)^39^ test to compare the performance of different models and investigate whether the population mean vectors are the same. The null hypothesis *H*_0_ indicates that there is no significant difference among the group means, which can be formulated as *H*_0_: *µ*_1_ = *µ*_2_ = *· · · = µ*_K_. We fail to accept *H*_0_ if the *p*-value for an experiment is less than the selected significance level (*α*) of 0.05, which implies that at least one group mean is significantly different from the others. The *F*-test rejects *H*_0_ at level *α* if

2 $$F = \frac{{S_1^2}}{{S_2^2}} > F_{K - 1,\,N - K}(\alpha )$$

3 $$S_1^2 = \mathop {\sum}\limits_{i = 1}^K {n_i(\bar Y_{i \cdot } - \bar Y)^2} /(K - 1)$$

4 $$S_2^2 = \mathop {\sum}\limits_{i = 1}^K {\mathop {\sum}\limits_{j = 1}^{n_i} {(Y_{ij} - \bar Y_{i \cdot })^2} } /(N - K)$$

where $S_1^2$ is the between-group variance and $S_2^2$ is the within-group variance. $\bar{Y}_{i}$ denotes the sample mean in the *i*th group, *n*_*i*_ is the number of observations in the *i*th group, $\bar Y$ is the overall mean of the data, and *K* denotes the number of groups. *Y*_*ij*_ is the *j*th observation in the *i*th group of *K* groups, and *N* is the overall sample size.

### Online cultivar recognition system

**Fig. 3:**
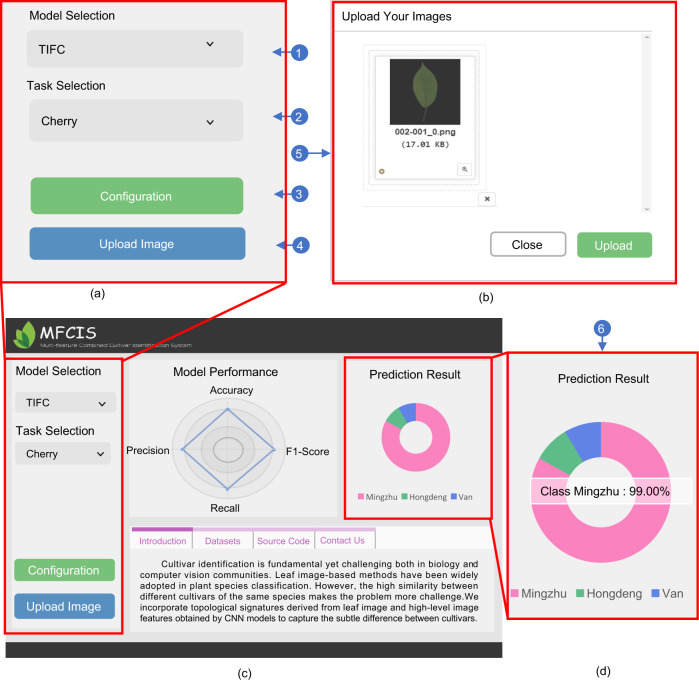
The main parts of the online cultivar recognition system. **a** Model and dataset selection panel. **b** Image preview and upload pop-up. **c** Main interface. **d** Results panel. The number in the blue circle shows the work step of the pipeline

To facilitate the adoption of the proposed pipeline, we developed an online cultivar recognition system (Fig. 3) on a publicly available website at http://www.mfcis.online. The website was developed based on the web framework Flask and is accessible via the internet. It provides an easy-to-use cultivar identification service that consists of the following three steps: (1) Model and dataset selection. In this step, users need to choose a preferred model and the target dataset and then wait for model loading and configuration. (2) Image uploading and cultivar recognition. In this step, users should select and upload a local image file via a pop-up. The chosen image can be enlarged to view the image details. Users should wait for several minutes until the prediction is made. (3) View the prediction result. The top 3 cultivar prediction results are displayed as a pie chart. Users can click the pie chart to view the prediction score of each cultivar. A detailed user manual can be found in the Supplementary Manual.

## Results

### Leaf morphological feature extraction by PH

**Fig. 4:**
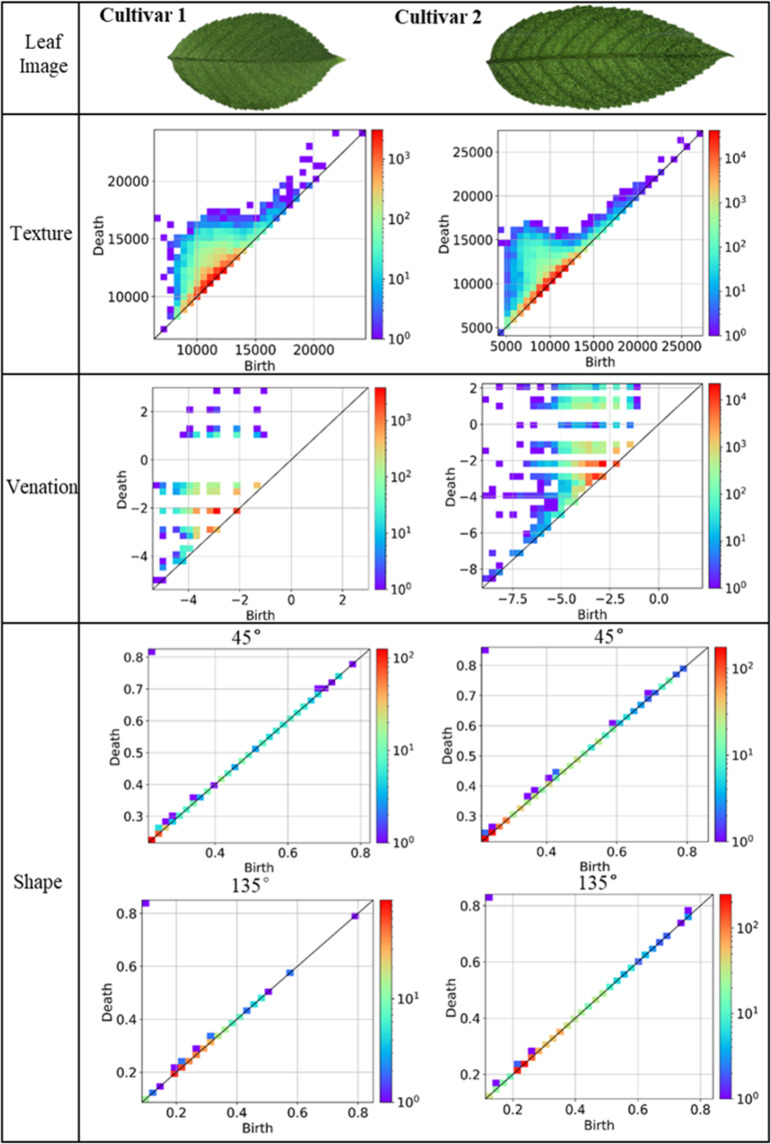
Leaf texture, venation, and shape PDs. Each column represents a sweet cherry cultivar. PD_0_ of the leaf texture and venation and PD_0_ of the leaf shape near 0°, 45°, 90°, and 135° are listed. All the diagrams are 2D histograms of points that discretize the plane into a grid of 32 × 32, and the point number of each grid is counted. The color of each grid represents the number of points

The leaf shape, texture, and venation PD samples are listed in Fig. 4. Owing to the high resolution of leaf images, the texture and venation PDs contained a large number of points. To intuitively compare the differences among PDs, we visualized all the PDs by their 2D histograms. The plane was discretized into a mesh of 32 × 32, and the point number of each grid was indicated by its color. As shown in Fig. 4, most topological features in the texture and venation PDs were close to the diagonal, meaning that they had a similar distributions among leaves from different cultivars. However, the points far from the diagonal had different distributions. Even though it was difficult to describe in words, the neural network could excavate the clues behind it. The leaf shape PDs in each direction had fewer points than the texture and venation PDs, and most of them were close to diagonal. To capture the local shape details while retaining the shape context, we considered all the directions and stacked three consecutive directions as a set of shape features.

### Performance evaluation of MFCIS on the sweet cherry cultivar dataset

To assess the effectiveness of our MFCIS pipeline, we tested its performance on the sweet cherry cultivar dataset, which contains leaf images that were collected at one growth stage. The model was trained with 200 epochs, and the learning rate halved if the validation loss did not decrease in five consecutive epochs. The model with the smallest validation loss was selected as the final model. The ratios of the test and validation data were set to 0.3 and 0.1, respectively. RMSProp was chosen as the optimizer with an initial learning rate of 0.001. All the RGB leaf images were resized to 256 × 256 pixels. The leaf morphological and CNN-extracted image features were concatenated as a joint descriptor. The identification accuracies were examined using ten different train-and-test splits.

The MFCIS pipeline achieved a mean accuracy of 83.52% on the sweet cherry dataset (Table 1). To highlight the higher performance of the MFCIS pipeline, we tested two classic CNN models on the sweet cherry dataset, and the details of the parameter settings are presented in supplementary materials section 2. The fine-tuned Xception, which achieved the highest performance on several species classification datasets (Table S1), was trained and obtained an accuracy that was 17% lower than that of the MFCIS pipeline (Table 1). A DCNN model customized for an apple cultivar recognition task^20^ was also implemented and achieved an accuracy that was 50.97% lower than that of the MFCIS pipeline (Table 1). Collectively, these results show the superiority of the MFCIS pipeline for leaf image classification tasks on a relatively small dataset.

**Table 1:** Comparison of the classification accuracies of the different models on the sweet cherry dataset

Method	Accuracy (%)
IDSC + DP	10.91
HSC	16.47
PH	42.08
Customized DCNN for apple cultivar classification	32.55
Fine-tuned Xception	66.52
MFCIS (Our model)	**83.52**

Only the Top-1 accuracy is shown. The accuracies in bold are the results of the proposed method.

We also evaluated the accuracies of the cultivar identification models with leaf features extracted by some image processing-based methods to exhibit the advantages of high-level image features (Table 1). Their parameter settings are presented in supplementary materials section 2. Image processing-based methods, shape classification using the inner-distance (IDSC)^40^ and hierarchical string cuts (HSC)^41^, were also tested on the sweet cherry leaf image dataset and achieved much lower accuracies than the fine-tuned Xception and MFCIS methods. The IDSC method was integrated with dynamic programming (DP) to match the contour points. The accuracy of the IDSC+DP algorithm was 10.91%, and the accuracy of the HSC algorithm was 16.47%. The classification model using only leaf morphological features extracted by PH (referred to as the PH method in Tables 1 and 2) was tested and obtained an accuracy of 42.08%. Even though it achieved much higher performance than IDSC + DP and HSC, it was less accurate than both the MFCIS pipeline and the fine-tuned Xception pipeline.

**Table 2:** Mean accuracies of the different cultivar classification models using leaves from each period in the soybean dataset independently

Method	Accuracy (%)				
	R1	R3	R4	R5	R6
IDSC + DP	19.67	23.67	20.33	20.00	16.00
HSC	27.02	31.07	31.20	30.60	27.71
PH	16.70	17.15	18.20	17.25	12.70
DF-VGG16/LDA	18.87	22.50	19.90	15.90	13.37
Fine-tuned Xception	30.60	35.97	33.37	29.73	20.40
MFCIS (Our model)	55.90^*^	61.40^*^	61.37^*^	59.80^*^	44.87^*^

All the models were tested with ten different train-and-test splits. Only the Top-1 accuracy is listed. The accuracies in bold are the results of the proposed method. The superscript “*” denotes a significant difference (*P* < 0.05) between the proposed method and the other models using one-way ANOVA.

**Fig. 5:**
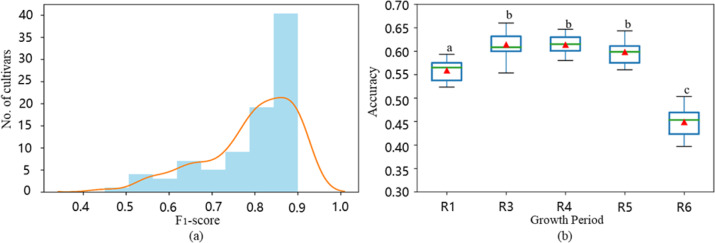
Cultivar identification results of different datasets. **a** The distribution of F_1_-scores on the sweet cherry dataset. **b** Boxplot of the accuracy of cultivar identification using leaves from each period in the soybean dataset independently. The triangle represents the mean accuracy of the corresponding period. The different letters on the top of each box indicate significant differences in accuracies among different growth stages (*P* < 0.05) using one-way ANOVA

To assess the confidence of the prediction results, we analyzed the final output of the softmax layer. The probability (the maximum value of the output of the softmax layer) of the correctly classified cultivars ranged from 0.26 to 1, and the mean probability was 0.96 with a standard deviation of 0.10. Furthermore, we explored the classification results of each cultivar by adopting the F_1_-score, which provided a more comprehensive evaluation of the predictions. The distribution of the F_1_-score in sweet cherry cultivar identification is shown in Fig. 5(a). Seventy-three cultivars had F_1_-scores higher than 0.7. The top-10 sweet cherry cultivars in terms of the F_1_-score were five commercial cultivars (“Luyu2”, “Windser”, “Regina”, “Luying 5”, and “Zayadka”) and five unreleased selections (“Zhao-B-4-5”, “Zhao-B-4-1”, “Zou-1”, “Zhao-B”, and “16-4”). All of them achieved F_1_-scores higher than 0.9, and more details are listed in Table S2. By observing the leaf images listed in Fig. S4, we found that these ten cultivars possess distinctive leaf shapes and margins. For example, the cultivar “Zayadka” has an ovoid shape with a sharper margin, while the cultivar “Luyu 2” has a smoother margin. Although these visible morphological differences could not be quantitatively measured, they could be easily captured by the CNN and summarized by high-level image features. Most of the poorly recognized cultivars have neither distinctive shape features nor distinctive leaf margin features. The large inner cultivar difference also played an important role in poor recognition performance.

### Performance evaluation of MFCIS on the soybean cultivar dataset

To evaluate the generalization performance of the proposed MFCIS pipeline, we applied it to a soybean cultivar dataset consisting of 5000 leaf images with 100 cultivars collected at five growth stages.

#### Cultivar Identification using leaves from each period independently

As shown in Fig. 1b, leaves of the same cultivar from different growth periods exhibit distinct shape, texture, and venation characteristics. To investigate the prediction ability of each growth period, we conducted the classification task using leaves of each period independently. In soybean cultivar identification, our pipeline was equipped with the same hyperparameters as those in sweet cherry cultivar identification.

Using MFCIS, the mean accuracies for the five growth stages ranged from 44.8 to 61.4% (Fig. 5b and Table 2). The R3 stage achieved a significantly higher accuracy than the other periods, whereas R6 achieved a substantially lower accuracy. The R1, R4, and R5 stages exhibited similar performances.

To highlight the superior performance of the proposed MFCIS pipeline, we tested the image processing-based methods IDSC+DP^40^, HSC^41^, and PH, as well as other deep-learning models, such as fine-tuned Xception^32^ and DF-VGG16/LDA^42^. Their parameter settings are presented in supplementary materials section 2. As shown in Table 2, the proposed MFCIS pipeline significantly outperformed all the other models across all the growth periods.

#### Cultivar identification using leaf images from all growth periods

To evaluate the impact of the intra-cultivar difference on cultivar identification, we mixed leaves from different periods within the same cultivar and conducted the classification task. All the parameter settings were the same as in the classification task using leaves from each growth period independently. The accuracies of using mixed leaves ranged from 52.6 to 56.5% in ten iterations with a mean accuracy of 54.45%, which was lower than that obtained when using each period independently, except for in the R6 stage.

#### Cultivar classification by score fusion

Although leaves exhibit considerable intra-cultivar differences across the five growth periods, the inter-cultivar discrepancies are substantial. In this experiment, we trained the model for each period independently and then applied a score-level fusion of the classification results from all five growth periods. Here, we fused the prediction scores of all periods with the same weights and evaluated the score fusion with ten different train-and-test splits.

The mean accuracy of the proposed MFCIS method was 91.4% with a standard deviation of 1.64 × 10^−2^, which is a considerable improvement compared to cultivar identification using leaves from each growth period independently. In addition, the proposed pipeline also significantly outperformed the fine-tuned Xception model without leaf morphological features, whose mean accuracy was 64% and standard deviation was 2.19 × 10^−2^.

### The online recognition platform

To date, the website can recognize sweet cherry and soybean cultivars. We also incorporated the Swedish and Flavia leaf image datasets for species classification since our model also achieved notable performance in plant species classification on these datasets (Table S1). We will continue to update the model to support more recognition tasks in the future. The website provides an intuitional process for cultivar identification. A detailed introduction is given in the supplementary video. However, to run the pipeline more efficiently and accurately, we highly recommend downloading the source code^43^ and running it on a high-performance server.

## Discussion

In crop breeding communities, cultivar identification has long been dependent on visual recognition by experts, which highly relies on personnel skills. Therefore, the accuracy cannot be guaranteed^44^. It is of great significance to speed up the recognition process and make it accessible for nonexperts^44^. With the development of deep learning and imaging techniques, leaf image-based cultivar identification studies have attracted attention from horticultural researchers and the computer vision society. Nevertheless, the accuracies of previous models remain a vital problem that has yet to be solved. This study proposed an effective strategy for leaf image acquisition, generated a highly accurate image analysis pipeline for cultivar identification, and constructed a web-based and user-friendly cultivar identification platform.

### Leaf-image acquisition strategy

We used the transmission scan technique, which is a low-cost and readily available method, to collect high-quality leaf images with subtle texture and clear leaf venation features. Similar to human fingerprints^45^, leaf texture and venation details provide vital information to discriminate cultivars^13,19,22^. However, they have not been widely applied thus far because venation and texture details in images obtained with general RGB cameras are usually not clear enough^46^.

The traditional way to improve the clarity of venation details was to treat the leaf with some chemical solutions before imaging, which destroys the leaf texture and is time consuming^47^. X-ray imaging techniques can obtain perfect venation details^46^, but they are expensive. Therefore, transmission scans are a better choice than snapshots by mobile devices and reflection scans to record leaf texture and venation details. In our experiments, we used a 16-bit depth grayscale image to extract the delicate texture and venation details of a leaf. Although we captured subtler texture changes, the computation of the pipeline was expensive. Hence, we recommend using grayscale-level compression to reduce the computational cost if fine texture features are unnecessary for image analysis tasks.

### Contributions of MFCIS to cultivar identification accuracy improvement

Compared to other image processing^12–19,22,40,41^ and classic machine learning-based algorithms^20,42,44^, the MFCIS pipeline significantly improved cultivar identification accuracy on both the soybean and sweet cherry leaf image datasets. One of the main reasons is the adoption of a feature fusion strategy that combines leaf morphological features extracted by PH and high-level image features extracted by deep learning. The combination of a CNN and PH might strengthen the generalization of the model when handling small datasets, which was reflected in the large improvement in accuracy obtained by MFCIS compared to the accuracy obtained by PH or a CNN independently in soybean cultivar recognition.

Another important reason is the adoption of a score-level fusion strategy that improved the utilization of information derived from leaves of the same cultivars collected from different growth periods. Considering that leaf morphology may be deformed during plant growth stages, we generated a soybean cultivar dataset with leaf images from five growth periods. Soybean leaves from each period were used independently to obtain identification accuracies varying from 44.87% (R6 period) to 61.37% (R3 period) in the MFCIS pipeline. Similar trends were also observed in other cultivar identification algorithms (Table 2). The accuracy difference across growth periods suggests that the morphological and venation characteristics inconstantly change during growth, which indicates the necessity and rationality for using leaves collected from multiple growth periods in cultivar identification models. On the other hand, the lower identification accuracy of the R6 growth period was probably due to the lower quality of the leaf images compared to the leaf images from the other growth periods. Wormholes and disease spots were observed in leaves from the R6 period, indicating that environmental conditions could be an important factor affecting leaf data quality and the inconsistent accuracy of plant variety identification across growth periods.

Moreover, two strategies that incorporated leaves of many growth periods were evaluated. (1) We trained the model using leaf images from all growth periods. (2) We trained the model for each period independently and then applied a score-level fusion of the identification results from all five growth periods. As expected, the multigrowth-period score fusion method brought a significant performance improvement compared to using mixed leaves from different periods within the same cultivar. This is mainly due to score-level fusion, which provides moderately rich information^48^ while avoiding dimension explosion and complicated dimension reduction^49^, which are difficult challenges encountered when using small datasets. The high accuracy of the multigrowth-period score fusion method proved that leaves from different growth periods of the same cultivar render complementary information for cultivar classification^38^.

### User-friendly online platform

Software is crucial for biological research^50^ and has a significant impact on research efficiency. Many well-designed software programs, such as Leaf GUI^7^, Rosette Tracker^8^, Leaf-GP^9^, MorphoLeaf^10^, and Phenotiki^11^, have been developed and have benefited biological researchers. However, since most of the software programs are offline clients, they usually require a complicated setup and a high-performance computer. We constructed a web-based cultivar recognition platform that is accessible to researchers and farmers through the internet. Compared to the existing software, the only requirement of our system is a browser and the internet, which is much more convenient.

Another user-friendly property of our online platform is full automation. Some leaf image-based cultivar identification pipelines, such as the multi-orientation region transform^22^, require the manual annotation of leaf images before fitting into the models. However, for our online platform, users can directly input the leaf images into the pipeline and output cultivar identification results without any manual processing of the input images.

### Comparison with molecular marker-based technologies

Our proposed system, including leaf-image acquisition, the MSCIF algorithm, and the online platform, provides an easy-to-use, simple and nondestructive method for plant variety identification based on the morphological differences in plant leaves. It is a rapid workflow with a total time cost of <15 min from scanning the leaf to obtaining the identification result. It can be arranged for outdoor-environment applications since the leaf image scanner and the online image processing algorithms are portable. The highly accurate results on the sweet cheery and soybean leaf datasets have proven the effectiveness of the pipeline. However, the morphological features of plant leaves are vulnerable to environmental and climatic conditions. Phenotypic plasticity is an inherent limitation in plant variety identification for all morphological descriptor-based methods. Identification accuracy depends on the similarity of the growth conditions between the training and testing leaf datasets. In addition to environmental factors, the growth period is another factor that may influence the generalization ability of morphological descriptor-based methods since leaves at different growth periods may exhibit various morphological features. If a model was trained on leaves from a certain period but used to predict leaves from the other growth periods, the accuracy would probably decrease significantly. Considering this growth period factor, we incorporated leaves across growth periods to train the MFCIS model on our soybean dataset.

In contrast, molecular marker-based technologies are known as powerful tools for the identification of plant varieties. Unlike leaf image-based methods that use morphological characteristics, they solely rely on variety-specific DNA fingerprints for discrimination. They, therefore, are more reliable and robust to different environmental conditions and the growth periods of plants. Furthermore, these methods do not need fully expanded leaves but can be applied in any growth stage using any plant tissues (such as seeds, fruits, and young leaves), making plant growth redundant^51^. With these advantages, molecular marker-based methods are being used in many breeding institutes and companies for routine identification in a wide range of ornamental, vegetable, fruit, and cereal crops^52–57^. However, their disadvantages are also apparent. First, molecular marker-based technologies are not universally usable since they lack specific primers, polymorphic molecular markers and referable results for many plant varieties^2^. Second, these methods are destructive for sample preparation, have low efficiency and may cause pollution due to the after-treatment of the chemicals^58^. Moreover, the cost is a major issue. Molecular markers are expensive to develop and require a well-equipped laboratory and skilled operational staff to use. The cost has greatly hindered the development and adoption of molecular marker-based techniques in many countries^59^. In conclusion, both leaf image- and molecular-based approaches have applicable scenarios and mutual complements for plant variety identification.

### Machine learning in horticulture research

Another aim of this work is to further machine learning-based plant phenotyping solutions in horticultural research, which is a fast-growing and multidisciplinary research area covering plant biology, advanced sensors, automation technology and big data analysis. The emergence of plant phenotyping in recent years has brought new perspectives to horticulture research. By combining advanced sensors and automation technology, more physiological and morphological traits across plant growth and development can be assessed, e.g., daily evapotranspiration^60^, crop biomass and leaf area indices^61^, seed germination^62^, and biophysical and biochemical traits^63^. In particular, it has been shown that many machine learning- and computer vision-based analytic methods improve phenotyping accuracy, reliability, and speed. However, there are some challenges that were encountered when applying machine learning-based algorithms in our study: (1) the performance of the learning algorithm could be poor if the phenotyping data is insufficient or has low quality; (2) many machine learning models, especially deep neural networks, are difficult to interpret and cannot further improve model performance. Hence, one of the major bottlenecks for plant phenotyping currently is the lack of powerful machining learning- and computer vision-based algorithms to make sense of phenotyping data effectively and accurately.

### Limitations and further work

In the MFCIS algorithm, leaf shape and margin features usually significantly impact cultivar identification^12^. For example, all the top-10 sweet cherry cultivars with the highest F_1_-scores had distinctive leaf shape and margin features. However, some poorly identified cultivars with lower F_1_-scores might be distinct in neither leaf shape nor margin features. Although the venation and texture features might produce a marked effect, the inner cultivar difference still made the proposed method fail to recognize some of them. Visualization of feature maps in the MFCIS model may help to detect more discriminative features^5^. The most widely used visualization techniques include deconvolution^64^, activation maximization^65^, and saliency maps^66^. For our study, each layer of the CNN had multiple convolutional kernels, and the sizes of the feature maps of deeper convolutional layers were small. These factors made it difficult to detect the potential classification clues by visualizing each filter. A saliency map can be used to identify the relatively important image pixels by computing the gradients of the output against the input image. To locate the vital leaf regions and detect discriminative features from these regions, we generated saliency maps of the misclassified and correctly classified leaves from the same cultivars, as shown in Fig. S5. However, we failed to locate the important leaf areas by comparing their saliency maps because the leaf regions with high saliency values varied in correctly classified and misclassified cases. It is essential to point out that the saliency maps shown in Fig. S5 were generated only from CNN-based feature extraction. We were unable to visualize the topological features with a saliency map. However, the accuracy improvement of the proposed MFCIS method was mainly due to the integration of topological features. Therefore, our future work for interpreting the results and improving the model accuracy will explain the impact of topological features on leaf image classification and propose a quantitative method to assess the contributions of each leaf feature.

When analyzing the soybean leaf image dataset, we developed a multigrowth-period score fusion strategy to utilize the time-series information incorporated in leaves of the same cultivars across growth periods. Although exciting accuracy was achieved, it is worthwhile to explore other data fusion techniques^67,68^. Chitwood and colleagues^69^ recently reported a composite leaf-modeling method for formulating leaf homologous universal landmarks by their relative positions in shoots over 4 years. It generated composite leaves to capture the spectrum of possible leaf morphologies for cultivar identification. This feature-level fusion strategy may achieve better performance than our score-level fusion approach because the weights of leaf features derived from different growth periods can be optimized during the training process.

## Conclusions

In this study, we proposed an automatic leaf image-based plant cultivar classification pipeline called MFCIS. Persistent homology was adopted to extract the morphological features of leaf shape, texture, and venation details. A fine-tuned deep CNN, Xception, was employed to extract features from leaf images. The task-optimal weighted leaf morphological features and high-level image features were automatically captured and concatenated in the training process. We obtained promising results for both sweet cherry and soybean cultivar recognition, illustrating the superiority and generalization capacity of the PH and CNN combination strategy for leaf image-based classification applications. A large performance improvement was observed when applying score fusion of different soybean growth periods, indicating that cultivar identification based on leaf morphological features requires the use of leaves collected at different growth periods to improve the identification accuracy.

## Supplementary information

Supplementary Materials

Supplementary User Manual

## Data Availability

The cherry and soybean image datasets are available at http://mfcis.online/. The environment is packaged as a Docker image to facilitate deployment and remove obstacles among different operating systems. All the code and Docker files are available at the source code repository.

## Code availability

Project name: Multifeature combined plant cultivar identification system

Project home page: https://github.com/WeizhenLiuBioinform/mfcis

Web-based platform: http://mfcis.online/

Source code: https://github.com/WeizhenLiuBioinform/mfcis

Operating system(s): Platform-independent

Programming language: Python 3.6

Requirements: Keras, TensorFlow, Scikit-image, Homcloud, etc. Detailed requirements are available at https://github.com/WeizhenLiuBioinform/mfcis/blob/master/requirements.txt

License: BSD-3-Clause available at https://opensource.org/licenses/BSD-3-Clause
