# Supplementary material for: MFCIS: an automatic leaf-based identification pipeline for plant cultivars using deep learning and persistent homology
Source: Hortic Res. 2021 Aug 1;8:172. doi: 10.1038/s41438-021-00608-w (PMC8325680; doi:10.1038/s41438-021-00608-w)
Supplement: Supplementary file 1 — Supplementary Materials [file 41438_2021_608_MOESM1_ESM.docx]

**Supplementary Materials**

**1. Leaf Morphological Features Extraction Using Persistent Homology**

**Leaf Shape** Inspired by Hofer et al. ^[1]^ and Turner et al^[2]^, we used PH to extract the leaf shape features. The binary leaf image was used as the input. Pixel values of leaf and background were set to be 1 and 0, respectively (Fig. S2(II a)). Shape features were captured by a multi-directional height function filtration. At first, the simplicial complex *K_0_* and *K_1_* were constructed. *K_0_* consisted of all the pixels belonging to the leaf. *K_1_* was made up of the 1-simplex [*p_0_, p_1_*] if *p_0_* and *p_1_* were four neighbors on the pixel grid. Then, the complex was filtrated from 30 different directions ^[1, 2]^ (Fig. S2(II b)). The different filtrated complex was obtained in the filtration procedure. As shown in Fig. S2(II c), the color of the image represents the pixel value. From purple to yellow, the value increases gradually. PD was computed for these filtrated complexes (Fig. S2(II c)). We also considered PDs of three consecutive directions as a set of features ^[1]^, which investigates the shape context information.

**Leaf Texture** Grayscale images were used as input, which is regarded as a real-value function $f\left( x,y \right), f\in[0, 65,535]$. The function value is the grayscale value of the image at position $(x,y)$. The grayscale value varies in different regions of the same leaf image, as shown in Fig. S2(III a). The red color shows a large grayscale value, while the blue shows a small grayscale value.

We constructed a sublevel-sets $\mathbb{F}^{\beta}=f^{-1}\left( -\infty, \beta\right], \beta\mathbb{\in R}$ as $\beta$ goes from $-\infty$ to $+\infty$. Each sublevel set was mapped to two vector spaces. The first vector space was the *0^th^* homology group generated by components of the sublevel sets and written as *H_0_*. The other was the *1^st^* homology group generated by loops of the sublevel sets and written as *H_1_*.

$f\left( x, y \right)=\left\{ \begin{aligned} 0 , &f(x,y)< \beta\\ 1 , &f(x,y)\geq\beta\end{aligned} \right.$ (1)

As the threshold increases, a set of time-series globally binarized images were generated, which could be described intuitively as Fig S2(III b). As the plane passed from low to high, components on the plane appear, grow, and merge, and the holes appear, shrink off, and disappear. These changes were recorded as the points in PD_0_ and PD_1_, as shown in Fig. S2(III c).

**Leaf Venation** Due to the high quality of the leaf images and the high contrast of the grayscale value between leaf venation and mesophyll (Fig. S2(IV a)), we adopted an adaptive threshold binarization algorithm, Sauvola, to segment the venation from enhanced leaf image ^[3]^, as shown in Fig. S2(IV b).

Unlike the leaf texture feature extraction, using grayscale images as input, the leaf venation feature extraction used the binary leaf venation images (Fig. S2(IV b)). A distance transform was applied to the leaf venation images at first. The distance between each background pixel and the nearest venation pixel was regarded as the value of the background pixel, and the distance between each venation pixel and the nearest background pixel was regarded as the value of the venation pixel, with the former represented as a positive number and the latter as a negative number (Fig. S2(IV c)). The sublevel-sets were constructed, and the filtration was applied as the distance increasing from $-\infty$ to $+\infty$. As the plane passed from low to high [Fig. S2(IV d)], the PH recorded the “birth” and “death” of the components and holes, as shown in Fig. S2(IV e).The PDs of texture and venation were computed by the Python package Homcloud [Ippei Obayashi, Hiraoka lab (AIMR, Tohoku University)], and the PDs of shape were calculated by the package Pershombox ^[1]^.

**2. Parameter Setting for Other CNN and Image Processing-based Models**

Here, we introduced the parameters of the methods listed in Table 1 and Table 2. For Shape Classification Using the Inner-Distance(IDSC)^[4]^ and Hierarchical String Cuts (HSC)^[5]^, we adopted the recommended parameters in the original work. The dynamic programing (DP) was chosen to matching contour points for IDSC. We trained the DCNN model customized for an apple cultivar recognition task on sweet cherry and soybean datasets using the same setting configuration as the original work ^[6]^. The fine-tuned Xception model was tested on the sweet cherry and soybean dataset. The stochastic gradient descent (SGD) was used as the optimizer for the fine-tuned Xception, and the other settings were the same as the default parameters in Keras. These settings were the same as those used in species classification (Table S1). The parameters for DF-VGG16/LDA^[7]^ on soybean dataset was set the same as the default parameters in Scikit-learn.

**Supplemental Figures:**

**
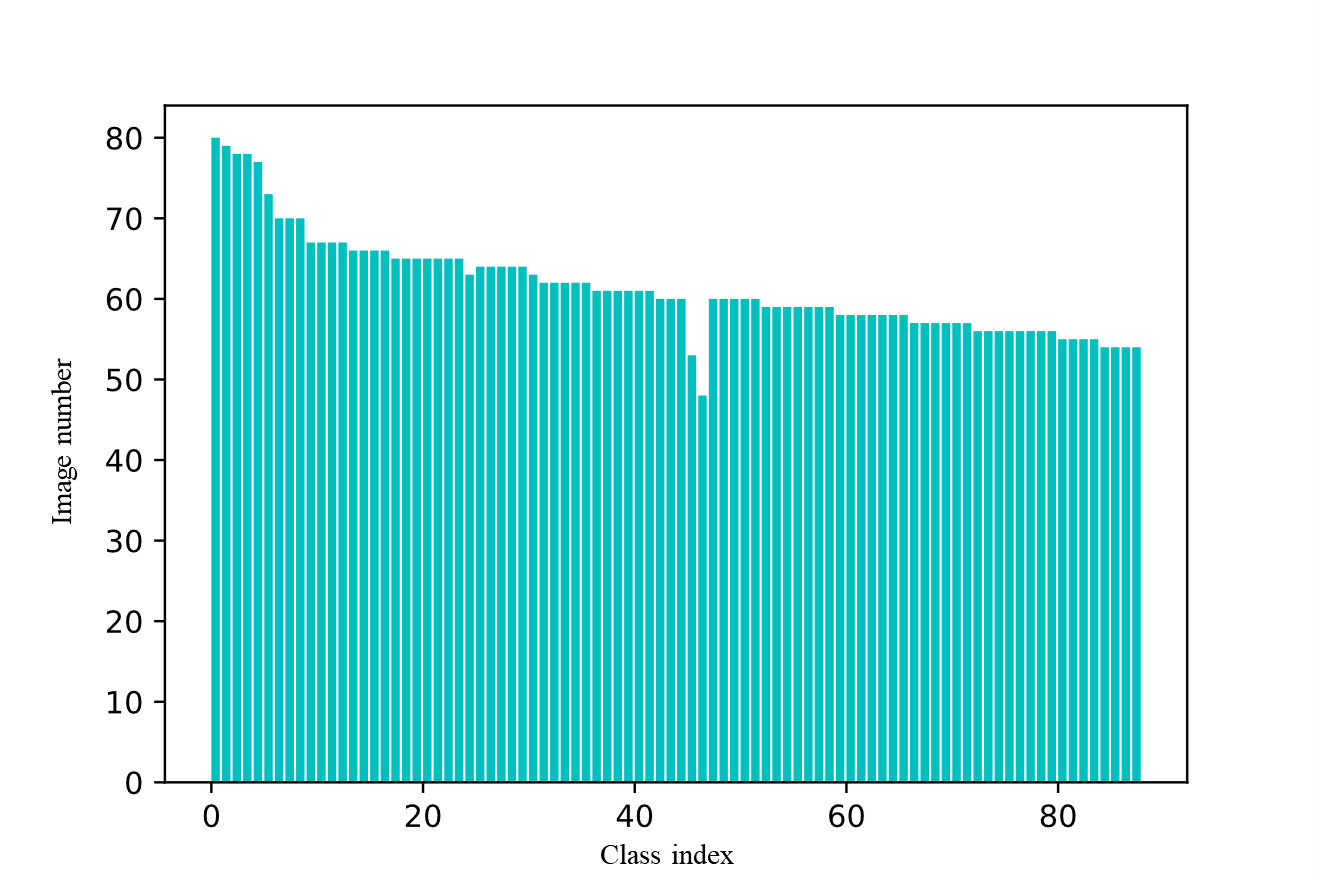
**

**Fig. S1 The image number of each sweet cherry cultivar.**


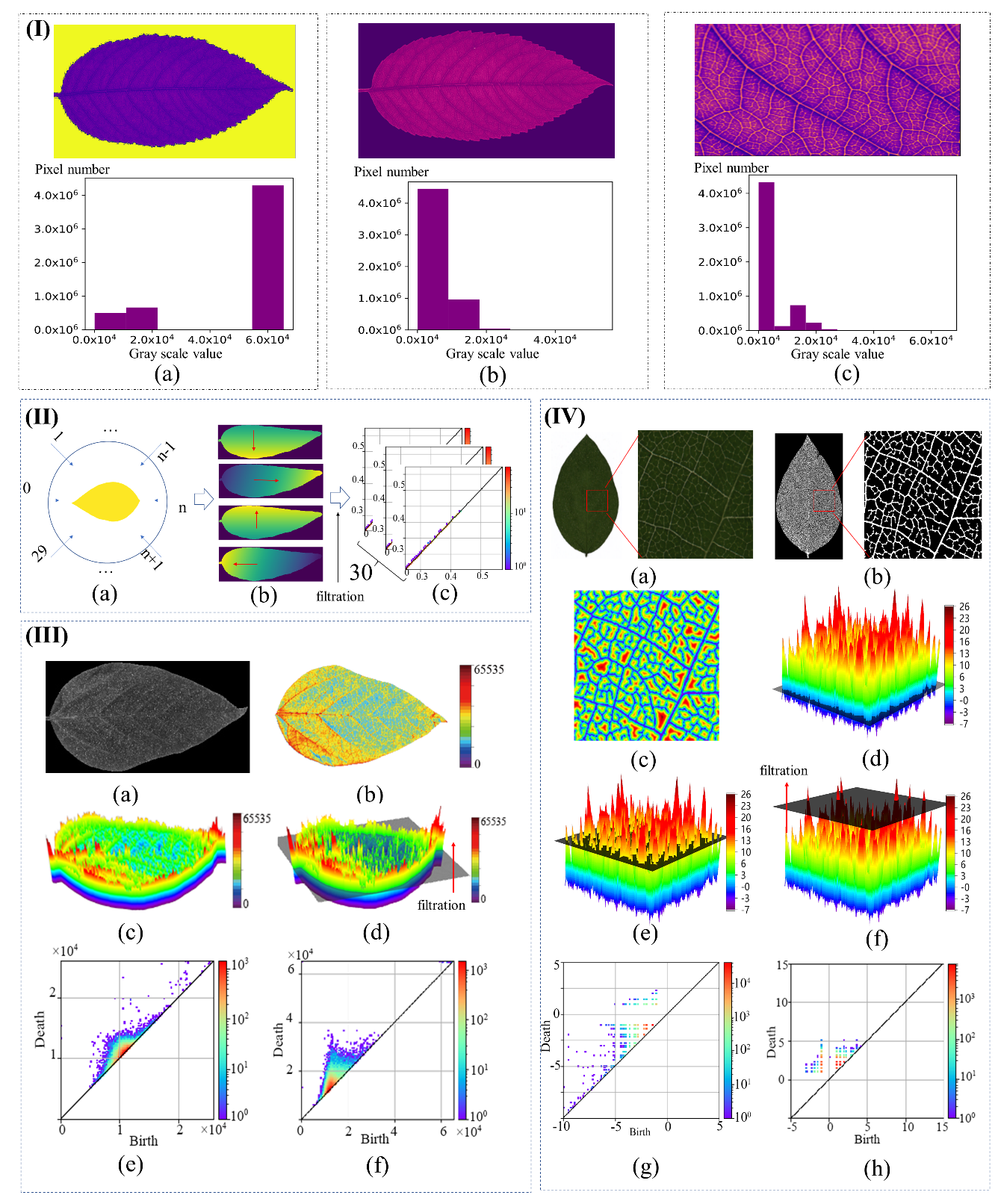


**Fig.S2 The procedure of leaf morphological features extraction using persistent homology.** **(I)** The procedure of image preprocessing. (a), (b), and (c) is the original grayscale image, background removed image, contrast-enhanced image, and their gray-level histogram, respectively. **(II)** The procedure of shape feature extraction. (a) Binary leaf mask image. (b) Height function filtration from 30 different directions. (c) Filtrated complex. (d) The first dimension PDs of 30 directions. **(III)** The procedure of texture feature extraction. (a) Grayscale value distribution heatmap. (b) Grayscale value function filtration. The red arrow indicates the filtration direction. (c) *0^th^* and *1^st^* dimension PD. **(IV)** The procedure of venation feature extraction. (a) Leaf image and the zoomed-in local details. (b) Venation segmentation results. (c) Distance transform results. (d)Distance function filtration. The red arrow indicates the filtration direction.


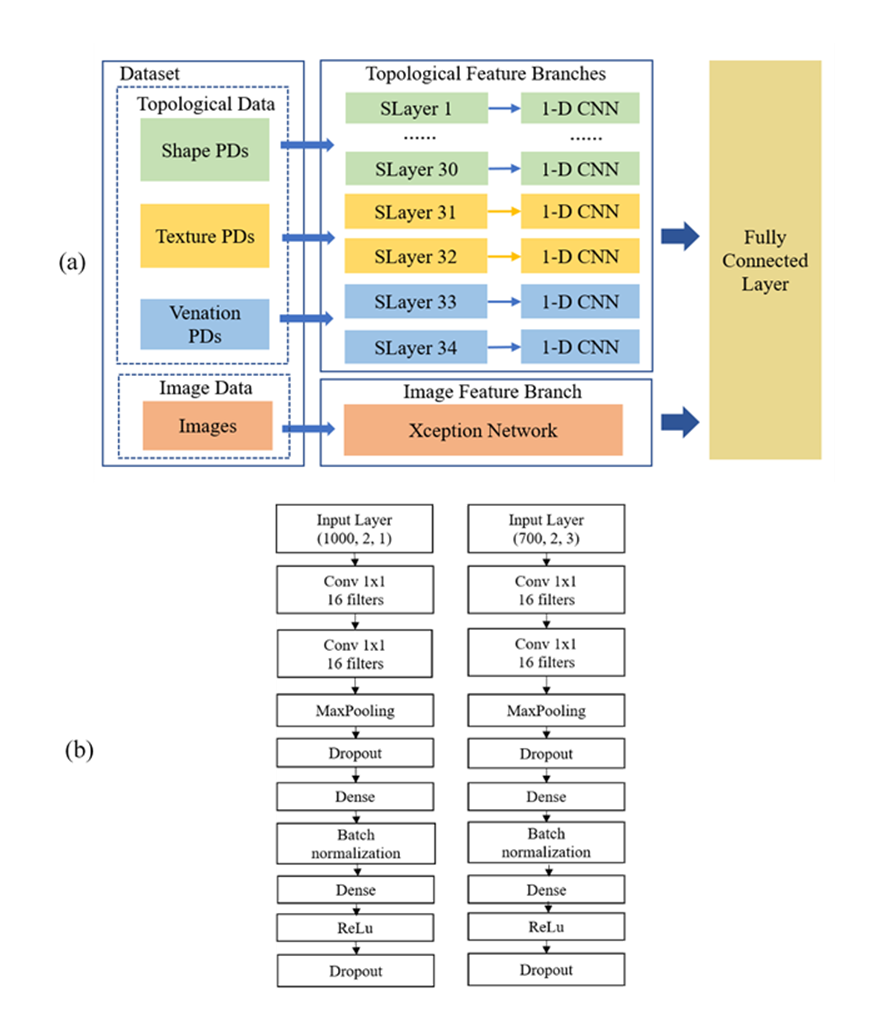


**Fig.S3 The model structure of the MFCIS pipeline.** (a) the whole model structure of this pipeline. (b)the model structure of topological feature branches.The left branch is for texture and venation features. There are two branches for texture and venation features, respectively. The right branch is for leaf shape. There are 30 branches for 30 different filtration directions.


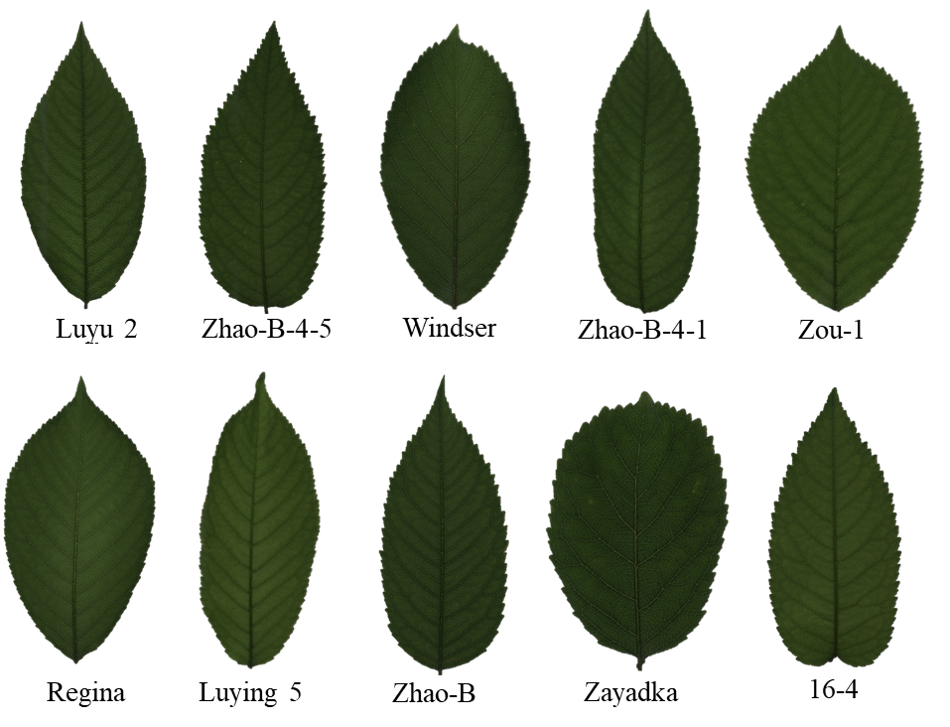


**Fig.S4 The top-10 sweet cherry cultivar leaf image with the highest recognition accuracy**


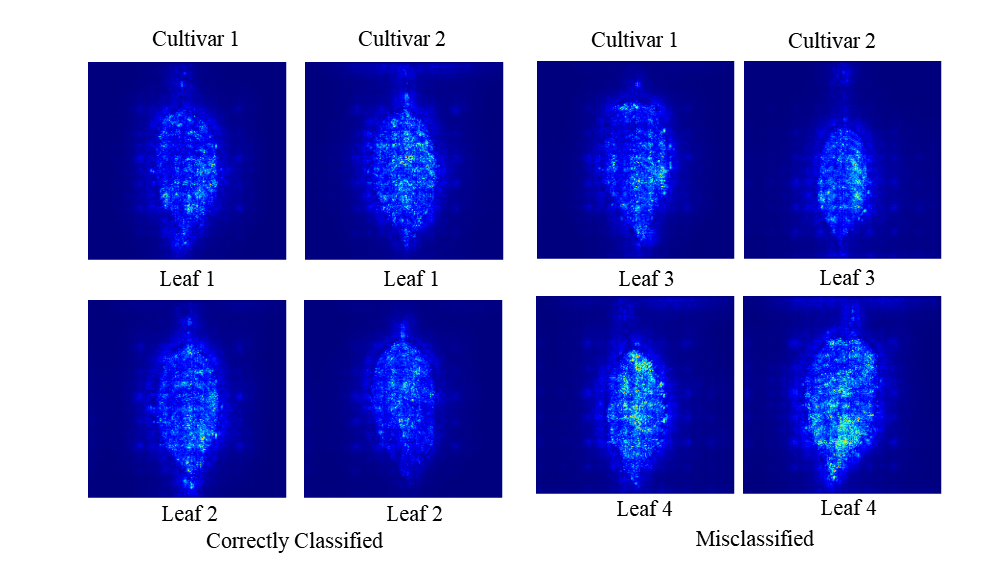


**Fig. S5 The visualization results of two cherry cultivars.**

**Supplemental Tables**

**Table.S1** Comparison of classification accuracy of the proposed MFCIS method to CNN-based methods on the Flavia and Swedish dataset.

| Dataset | Method | Accuracy (%) |
| --- | --- | --- |
| Swedish | Leaf Net ^[8]^ | 97.07 |
|  | CNN-RNN ^[7]^ | 98.80 |
|  | Fine-tuned Xception | 99.73 |
|  | **MFCIS (Our model)** | **99.85** |
| Flavia | Leaf Net ^[8]^ | 97.90 |
|  | DF-VGG16/LDA^[7]^ | 99.00 |
|  | Fine-tuned Xception | 99.30 |
|  | **MFCIS (Our model)** | **99.64** |

Note: Only the Top-1 accuracy is shown.

**Table.S2** The detailed results of sweet cherry cultivar recognition.

| Cultivar Name | Type | Precision | Recall | F1-score |
| --- | --- | --- | --- | --- |
| Zhao-B | Selection | 0.938 | 0.963 | 0.947 |
| Mingzhu | Cultivar | 0.904 | 0.898 | 0.899 |
| Black Gda | Cultivar | 0.910 | 0.932 | 0.918 |
| KN3 | Cultivar | 0.880 | 0.900 | 0.887 |
| Regina | Cultivar | 0.959 | 0.948 | 0.952 |
| Luying 3 | Cultivar | 0.901 | 0.893 | 0.895 |
| Luyu | Cultivar | 0.836 | 0.853 | 0.844 |
| Burlat | Cultivar | 0.806 | 0.795 | 0.795 |
| Xi-WS | Selection | 0.933 | 0.917 | 0.922 |
| 7-9 | Selection | 0.904 | 0.945 | 0.922 |
| Hedeifidcr | Cultivar | 0.888 | 0.825 | 0.851 |
| France skaia | Cultivar | 0.858 | 0.805 | 0.824 |
| Van | Cultivar | 0.464 | 0.605 | 0.521 |
| U3-2 | Selection | 0.814 | 0.795 | 0.802 |
| Sela XVan | Selection | 0.883 | 0.915 | 0.896 |
| Matuska | Cultivar | 0.820 | 0.760 | 0.785 |
| U9-6 | Selection | 0.818 | 0.740 | 0.768 |
| Samihuang | Cultivar | 0.787 | 0.865 | 0.821 |
| 3-1 | Selection | 0.879 | 0.890 | 0.882 |
| Zhao-B-4-1 | Selection | 0.938 | 0.985 | 0.959 |
| Zou-1 | Selection | 0.982 | 0.935 | 0.955 |
| Aticka | Cultivar | 0.648 | 0.785 | 0.705 |
| Sam | Cultivar | 0.769 | 0.810 | 0.784 |
| Sastina | Cultivar | 0.926 | 0.955 | 0.938 |
| 12-10 | Selection | 0.870 | 0.836 | 0.850 |
| Lapins | Cultivar | 0.867 | 0.805 | 0.831 |
| Santina | Cultivar | 0.721 | 0.695 | 0.706 |
| Hongdeng | Cultivar | 0.613 | 0.673 | 0.638 |
| 9-4 | Selection | 0.888 | 0.840 | 0.857 |
| Zaoganyang | Cultivar | 0.913 | 0.925 | 0.915 |
| SDL-1 | Selection | 0.902 | 0.905 | 0.901 |
| Yogal mini | Cultivar | 0.932 | 0.914 | 0.921 |
| Tieton | Cultivar | 0.831 | 0.809 | 0.817 |
| Skeena | Cultivar | 0.862 | 0.815 | 0.836 |
| Luying 8 | Cultivar | 0.899 | 0.959 | 0.927 |
| Zayadka | Cultivar | 0.914 | 0.974 | 0.941 |
| Luying 9 | Cultivar | 0.901 | 0.812 | 0.849 |
| Kolhoznaia | Cultivar | 0.685 | 0.717 | 0.698 |
| Sweet heart | Cultivar | 0.814 | 0.871 | 0.839 |
| Luzao | Cultivar | 0.917 | 0.914 | 0.912 |
| Fedora | Cultivar | 0.844 | 0.828 | 0.833 |
| Olenka | Cultivar | 0.795 | 0.910 | 0.847 |
| Do104 | Cultivar | 0.840 | 0.782 | 0.800 |
| Saylor | Cultivar | 0.660 | 0.689 | 0.667 |
| J98-96 | Selection | 0.821 | 0.782 | 0.798 |
| Luyu 2 | Cultivar | 0.985 | 0.983 | 0.983 |
| Luying 5 | Cultivar | 0.927 | 0.982 | 0.951 |
| Canada zaoshu | Selection | 0.896 | 0.920 | 0.905 |
| Kristiu | Cultivar | 0.706 | 0.634 | 0.660 |
| Sunburst | Cultivar | 0.666 | 0.656 | 0.657 |
| Zaodaguo | Cultivar | 0.782 | 0.832 | 0.802 |
| Summit | Cultivar | 0.900 | 0.948 | 0.920 |
| Linda | Cultivar | 0.896 | 0.843 | 0.862 |
| 16-4 | Selection | 0.955 | 0.932 | 0.940 |
| 16-3 | Selection | 0.838 | 0.882 | 0.855 |
| Corol | Cultivar | 0.811 | 0.865 | 0.833 |
| Zhuangyuanhong | Cultivar | 0.891 | 0.828 | 0.856 |
| Zhao-B-4-5 | Selection | 0.975 | 0.994 | 0.983 |
| Black Tartarian | Cultivar | 0.937 | 0.926 | 0.929 |
| 12-4 | Selection | 0.862 | 0.788 | 0.816 |
| Iran -1 | Cultivar | 0.808 | 0.741 | 0.767 |
| Ulster | Cultivar | 0.689 | 0.582 | 0.611 |
| GII -97 | Cultivar | 0.789 | 0.821 | 0.801 |
| DaHuang | Cultivar | 0.84 | 0.928 | 0.878 |
| Galina | Cultivar | 0.630 | 0.500 | 0.544 |
| Brooks | Cultivar | 0.885 | 0.839 | 0.861 |
| Proshalinaia | Cultivar | 0.931 | 0.857 | 0.892 |
| Fuchen | Cultivar | 0.772 | 0.66 | 0.703 |
| US-4-1 | Selection | 0.607 | 0.494 | 0.529 |
| Luying 2 | Cultivar | 0.554 | 0.584 | 0.560 |
| Hongmi | Cultivar | 0.756 | 0.722 | 0.734 |
| 7-4 | Selection | 0.787 | 0.577 | 0.652 |
| 6-10 | Selection | 0.667 | 0.565 | 0.604 |
| Luying 4 | Cultivar | 0.688 | 0.701 | 0.689 |
| Jindinghong | Cultivar | 0.872 | 0.875 | 0.869 |
| Yellow Glass | Cultivar | 0.649 | 0.672 | 0.654 |
| Atika-5 | Cultivar | 0.787 | 0.793 | 0.781 |
| 12-11 | Selection | 0.852 | 0.862 | 0.857 |
| Windser | Cultivar | 0.956 | 0.976 | 0.965 |
| Salah | Cultivar | 0.851 | 0.833 | 0.841 |
| Atika | Cultivar | 0.924 | 0.875 | 0.895 |
| US5-1 | Selection | 0.89 | 0.872 | 0.879 |
| Qizao | Cultivar | 0.860 | 0.806 | 0.830 |
| Rainier | Cultivar | 0.834 | 0.797 | 0.807 |
| Houdong1-5 | Selection | 0.711 | 0.819 | 0.755 |
| Mashad Black | Cultivar | 0.811 | 0.783 | 0.793 |
| Utach Giant | Cultivar | 0.525 | 0.481 | 0.494 |
| Hongnanyang | Cultivar | 0.786 | 0.655 | 0.707 |

**Supplementary Video**

The usage instruction of the online recognition platform is presented in Supplemental Video at <http://mfcis.online/static/introduction_video.mp4>.

# References

[1]C. Hofer, R. Kwitt, M. Niethammer et al. Deep learning with topological signatures, in: Advances in Neural Information Processing Systems, 2017: pp. 1634–1644.

[2]K. Turner, S. Mukherjee, D.M. Boyer. Persistent homology transform for modeling shapes and surfaces, Information and Inference: A Journal of the IMA. 3 (2014) 310–344.

[3]J. Sauvola, M. Pietikäinen. Adaptive document image binarization, Pattern Recognition. 33 (2000) 225–236.

[4] Ling H, Jacobs DW. Shape Classification Using the Inner-Distance. *IEEE Transactions on Pattern Analysis and Machine Intelligence* 2007; **29**: 286–299.

[5] Wang B, Gao Y. Hierarchical String Cuts: A Translation, Rotation, Scale, and Mirror Invariant Descriptor for Fast Shape Retrieval. *IEEE Trans on Image Process* 2014; **23**: 4101–4111.

[6] C. Liu, J. Han, B. Chen et al. A Novel Identification Method for Apple (Malus domestica Borkh.) Cultivars Based on a Deep Convolutional Neural Network with Leaf Image Input, Symmetry. 12 (2020) 217.

[7]A. Kaya, A.S. Keceli, C. Catal et al. Analysis of transfer learning for deep neural network-based plant classification models, Computers and Electronics in Agriculture. 158 (2019) 20–29.

[8] Barré P, Stöver BC, Müller KF et al. LeafNet: A computer vision system for automatic plant species identification. Ecological Informatics 2017; 40: 50–56.
