# Supplementary material for: MFCIS: an automatic leaf-based identification pipeline for plant cultivars using deep learning and persistent homology
Source: Hortic Res. 2021 Aug 1;8:172. doi: 10.1038/s41438-021-00608-w (PMC8325680; doi:10.1038/s41438-021-00608-w)
Supplement: Supplementary file 2 — Supplementary User Manual [file 41438_2021_608_MOESM2_ESM.docx]

**Supplementary User Manual**

## User Manual of MFCIS Online Platform

We provided an online platform of the MFCIS, the website address was <http://mfcis.online/>. Anyone can access the website through a web browser (Chrome is recommended). Guidance will be given to users when they visit the website the first time. And the guidance interface will help the user get familiar with the platform, as shown below:

1. Step 1: Model Selection

The user can select a model from the drop-down box. The Xception and TP+Xception (MFCIS) are supported now, and more models will be added in the future.


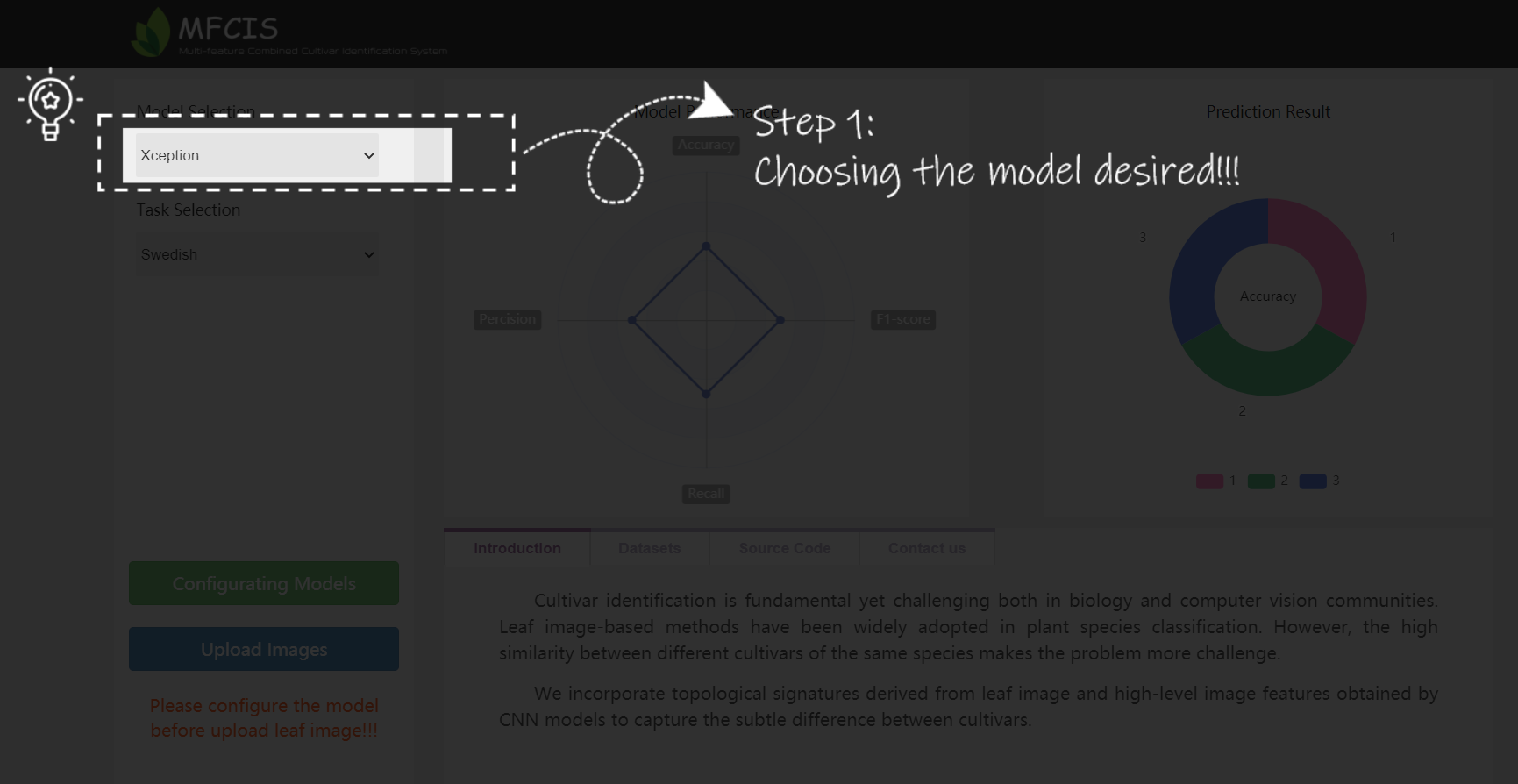


1. Step 2: Dataset Selection

Selecting a target dataset from the drop-down box. Four datasets are currently available: Swedish, Flavia, Soybean, and Sweet Cherry. If the Soybean dataset is selected, the user should choose a growth period by click the check box, as shown in Step 3.


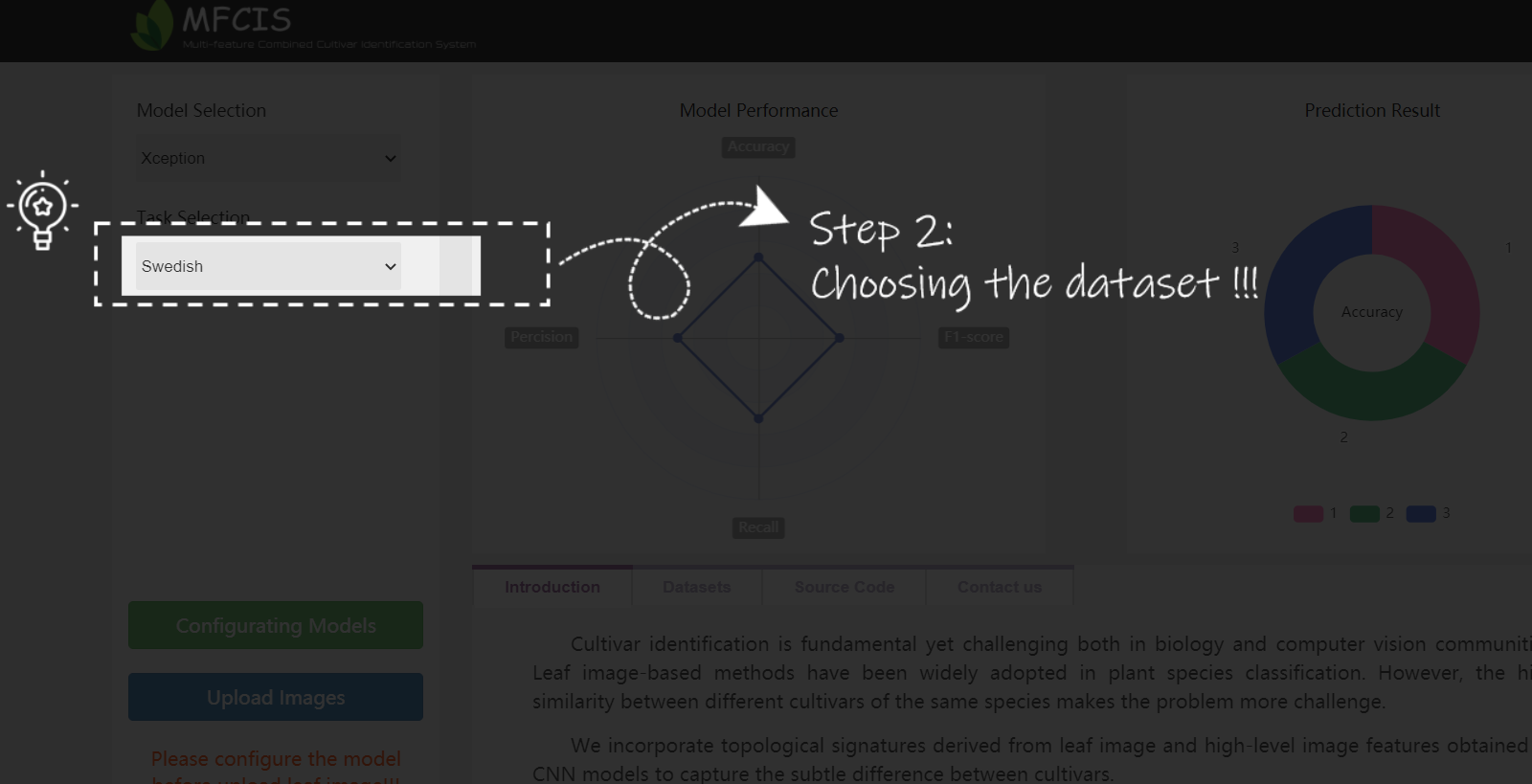


1. Step 3: Growth Period Selection (optional)

The growth period should be chosen when the soybean dataset is selected. If more than one growth period is selected, the platform will apply the score fusion of these growth periods.


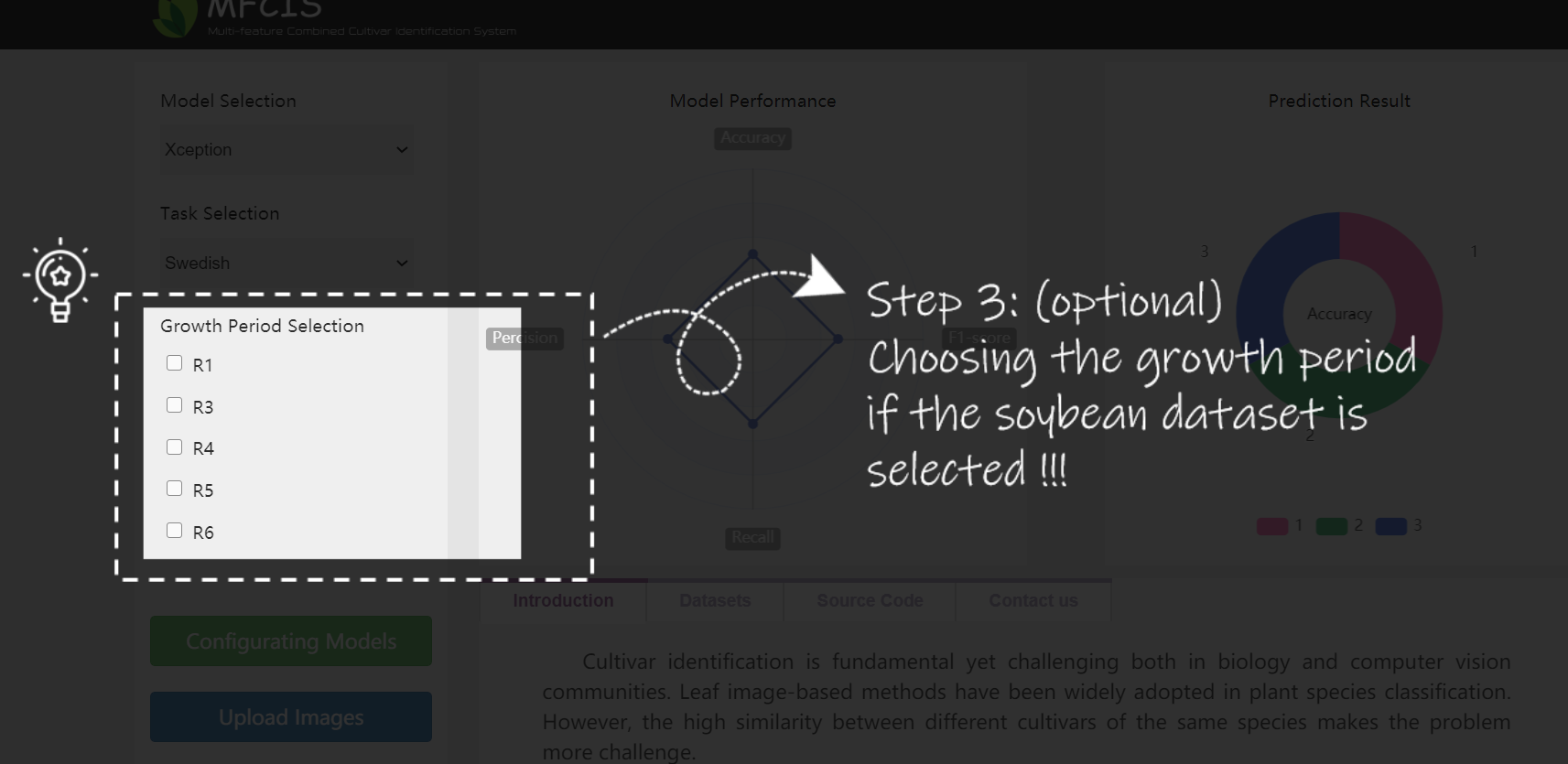


1. Step 4: Model Loading and Configuration

When the dataset and model are selected, the user should click this button to load and configure the selected model. This process may take a few seconds. The main interface will be locked until the model loading and configuration complete.


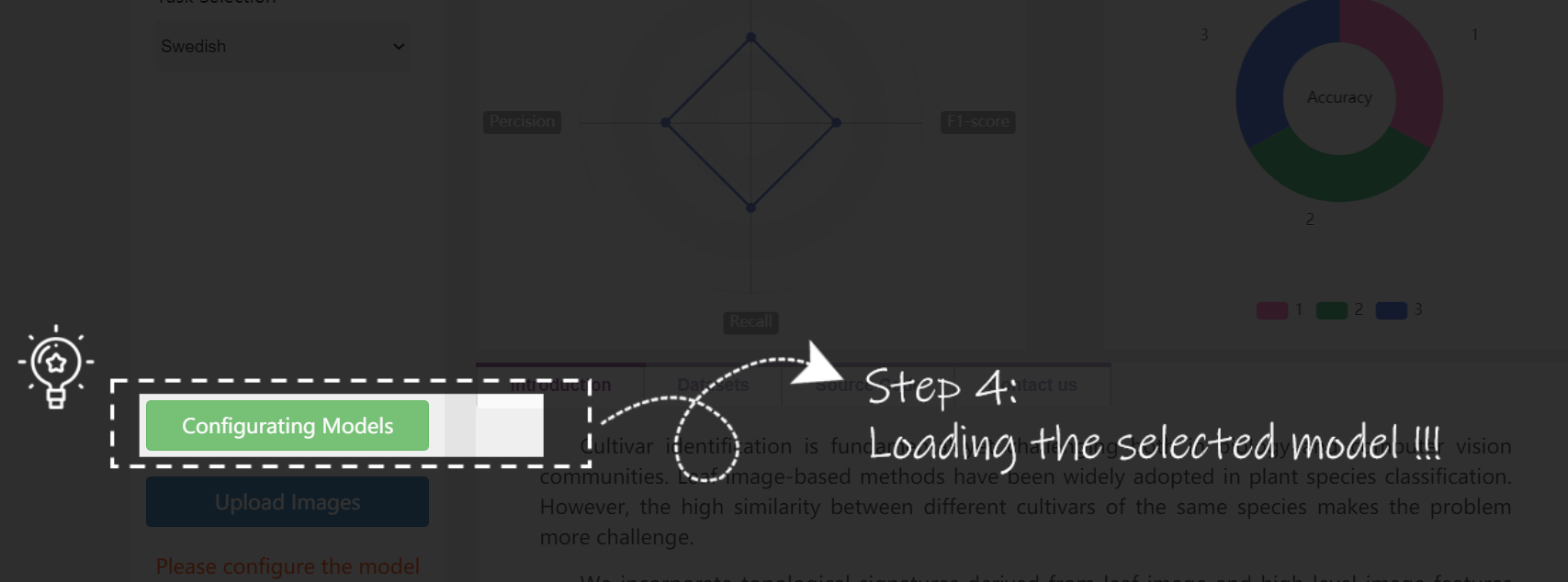


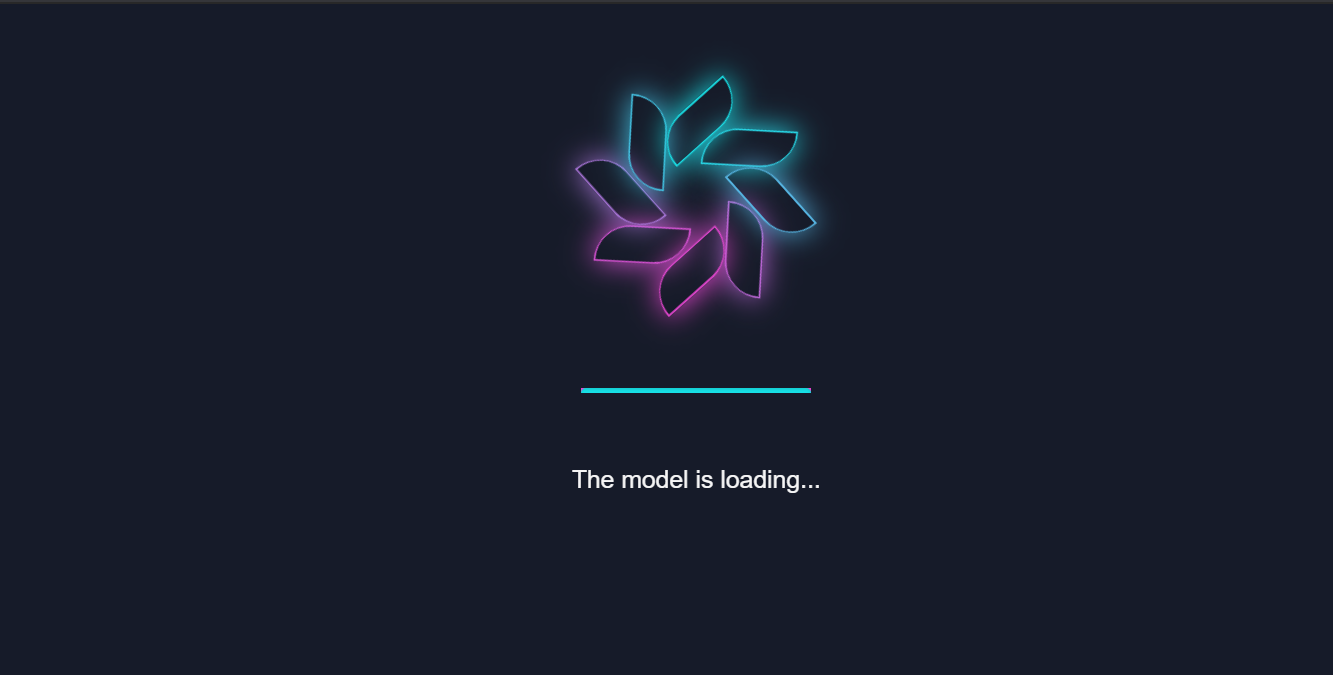


The waiting page.

1. Image Uploading

After loading and configuring the model, the user should upload the local images by clicking the “Upload Images” button.


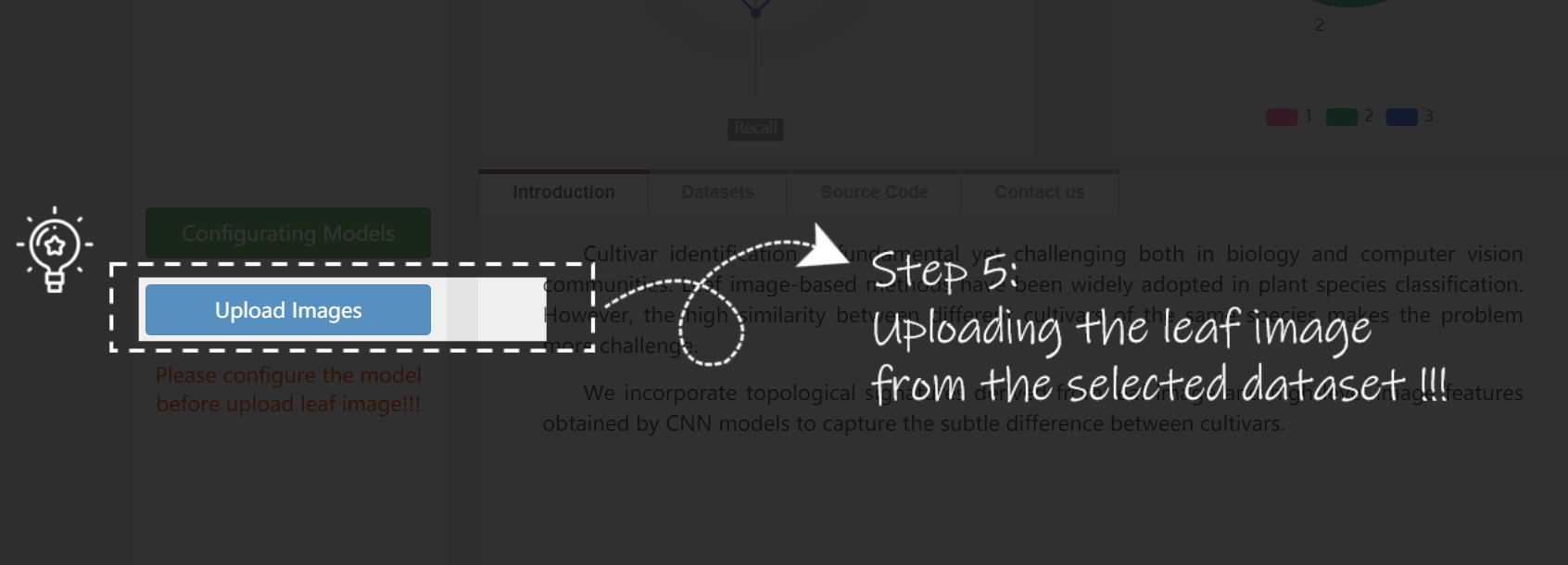


And then, the image uploading panel will pop up.


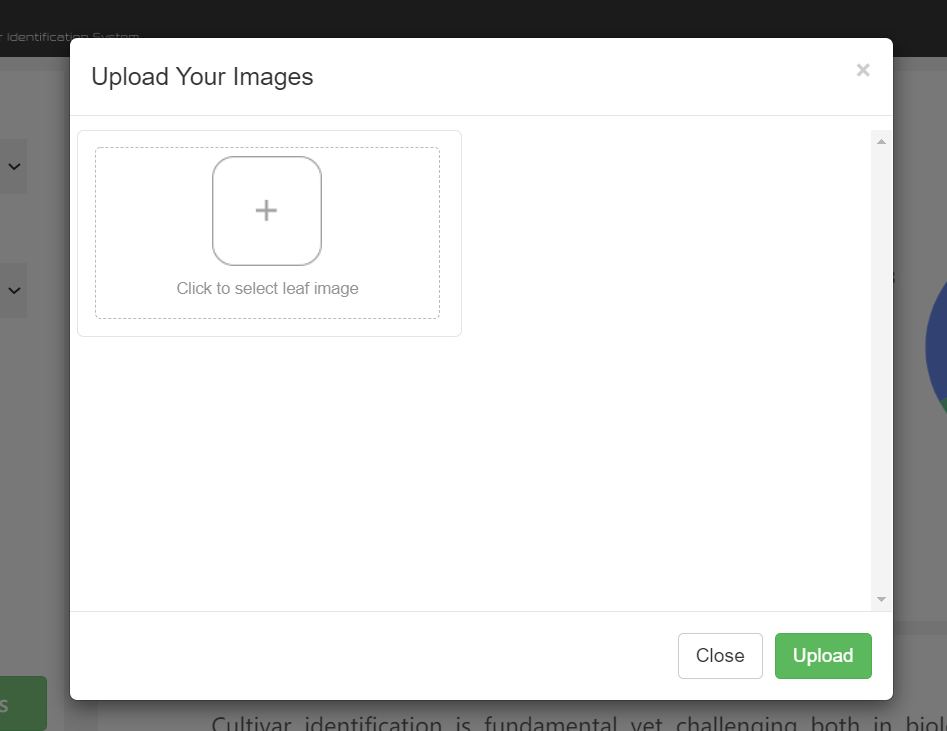


The image upload panel.


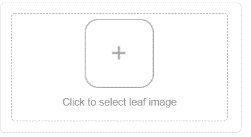


If the user clicks the icon a file selection panel will pop up,

and the local leaf images can be selected. Once the image is selected, the user can preview and reselect images, as below:


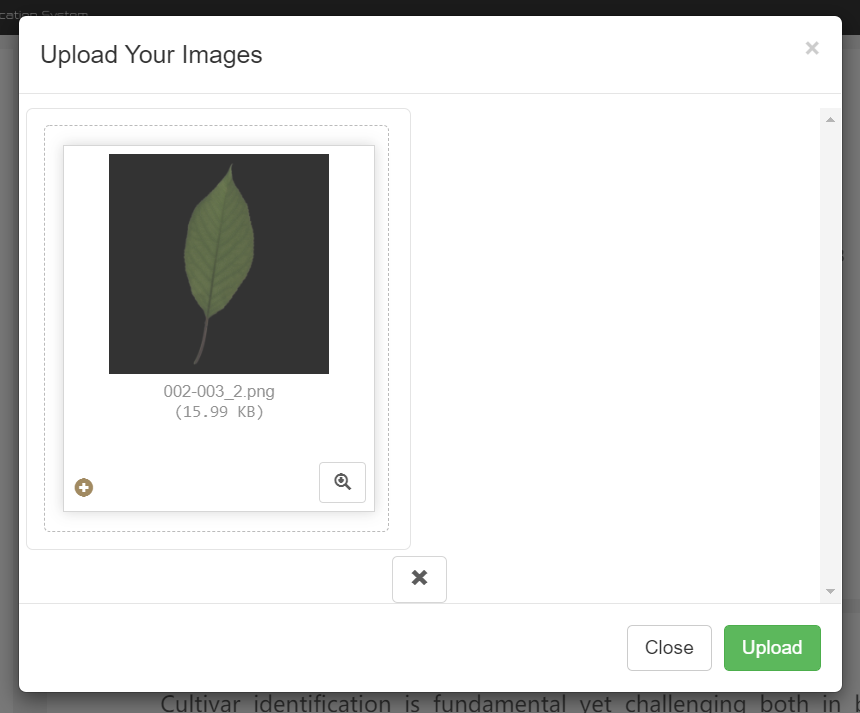


Then the upload button should be clicked to upload the image to the server and start the recognition. The recognition may take a few seconds for Xception model and a few minutes for TP+Xception model, since the computation of topological features is time-consuming. The page will redirect to the waiting page until the recognition complete.

1. Prediction Result

Once the recognition complete, the Top-3 results will be displayed by a pie chart.


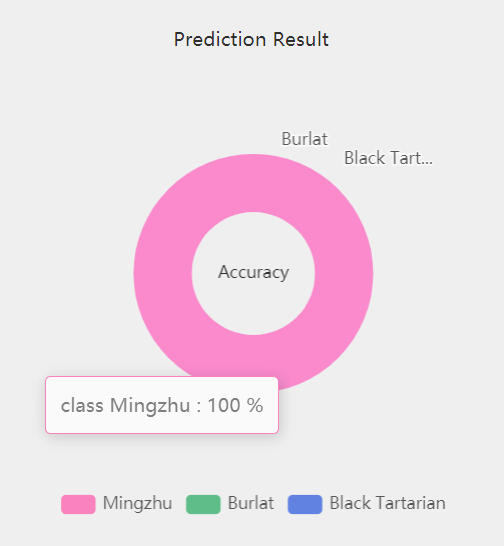


1. Dataset Download and Contact Information

The user can download the datasets and get the contact information of us in the following panel.


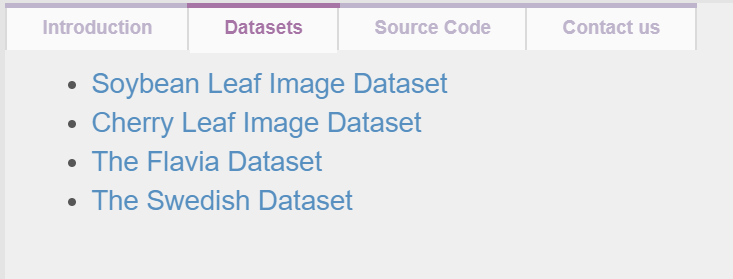


## The User Manual of Source Code

Besides the online platform, we also release the code of the proposed method, (<https://github.com/WeizhenLiuBioinform/mfcis>). The user who wants to transfer the method to any other species can download the source code and develop based on it.

The folder structure is shown as follows:

**
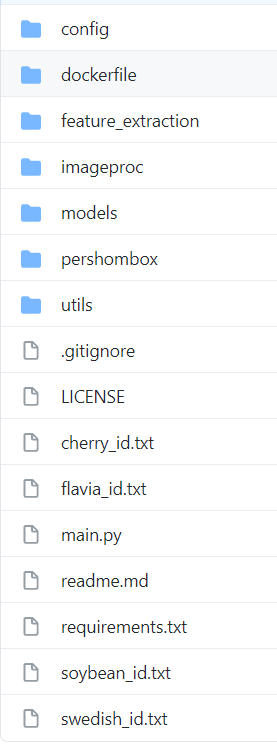
config:** The user can modify the model settings and dataset path in the model configuration file.

**dockerfile:** If the user wants to run the source code as a docker container. The folder provides a DockerFile to build the docker image.

**feature_extraction:** The folder provides the basic image preprocessing utils.

**models:** The file in this folder provides the model structure and construction API.

**pershombox:** The backend of computation of persistence diagram. (Hofer et al, NIPS 2017)

**utils:** Files in this folder provides the essential data loading util functions.

**requirements.txt :** The python package dependencies.

**xx_id.txt:** The label map file of the corresponding dataset. It maps the class name to the class index.

**main.py:** The entrance of the program. It implements the leaf image classification based on the modules introduced above.

For the detailed instructions, please refer to the comments in the source code and the main pages of the project (<https://github.com/WeizhenLiuBioinform/mfcis>) Please raise an issue on Github when you facing a problem in the usage.
